# *N*-Acetylcysteine Attenuates Amikacin-Induced Biochemical, Inflammatory, and Histopathological Alterations in Mice

**DOI:** 10.3390/biology15181618

**Published:** 2026-09-14

**Authors:** Lujain Khalid Khan, Mashal M. Almutairi, Mashan L. Abdullah, Abdullah S. Alali, Abdulmohsen K. Alanazi, Moureq R. Alotaibi, Homood M. As Sobeai, Asma F. Alotaibi, Fatin A. Alrashedi, Shakir D. AlSharari, Youssef Sari, Thikra Algahtani, Fawaz Alasmari

**Affiliations:** 1 Department of Pharmacology and Toxicology, College of Pharmacy, King Saud University, Riyadh 11451, Saudi Arabia; 2 Department of Experimental Medicine, King Abdullah International Medical Research Center (KAIMRC), King Saud Bin Abdulaziz University for Health Sciences (KSAU-HS), Ministry of National Guard-Health Affairs (MNGHA), Riyadh 11481, Saudi Arabia; 3 Department of Pharmacology and Experimental Therapeutics, College of Pharmacy and Pharmaceutical Sciences, The University of Toledo, Toledo, OH 43614, USA; 4 Centre for Pharmaceutical Medicine Research, Institute of Pharmaceutical Science, King’s College London, London WC2R 2LS, UK

**Keywords:** amikacin, *N*-acetylcysteine, inflammatory markers, biochemistry, light-dark box

## Abstract

**Background:** Amikacin is an effective aminoglycoside antibiotic, but its clinical use is limited by nephrotoxicity, hepatotoxicity, and neurotoxicity. *N*-acetylcysteine (NAC), a glutathione precursor and antioxidant, may help reduce these side effects. This study evaluated whether NAC attenuated selected amikacin-induced biochemical, inflammatory, and histopathological alterations in mice. **Methods:** Male Swiss albino mice were divided into four groups: control, amikacin, NAC, and amikacin-NAC, and the behavioral performance was assessed using the light-dark box test. Serum samples were collected for measurements of kidney and liver biomarkers, metabolic markers, and electrolytes (calcium, inorganic phosphate, and uric acid). A multiplex cytokine/chemokine panel was used to assess the effects of amikacin with/without NAC on systemic inflammation. **Results:** Amikacin-treated mice showed a reduction in the time spent in the light area, and this effect was improved by NAC. Amikacin also altered certain biochemistry parameters, increasing levels of aspartate transferase and albumin; these changes were attenuated by NAC. Amikacin caused marked increases in certain inflammatory cytokines and chemokines, including IL-6, IL-12p70, IL-22, IL-18, CXCL10, and CCL11. Treatment with NAC lowered IL-6, IL-12p70, IL-22, IL-18, CXCL10, and CCL11. The sparse partial least squares discriminant and heatmap analysis of the serum inflammatory profile revealed segregation of the amikacin group from the other three groups. Importantly, the histopathological assays on renal and cerebral cortex showed NAC caused improvements, with well-shaped tissues, attenuating the cortical histopathological alterations observed in animals treated with amikacin. **Conclusions:** These findings suggest that NAC may have the potential to attenuate certain biochemical, inflammatory, and histopathological alterations associated with high-dose amikacin exposure.

## Simple Summary

Amikacin is an effective antibiotic, but exposure to high doses may be associated with biochemical, inflammatory, and tissue alterations. This study investigated whether *N*-acetylcysteine (NAC) could attenuate some of these changes in mice exposed to a high dose of amikacin. Mice were treated with amikacin with or without NAC for five days, followed by assessment of anxiety-like behavior, serum biochemical parameters, inflammatory cytokines and chemokines, and histopathological changes in the kidney and cerebral cortex. Amikacin exposure caused anxiety-like behavior, increased aspartate transferase, albumin and circulating inflammatory mediators, and produced histopathological changes in the kidney and cerebral cortex. NAC treatment attenuated several of these alterations, including changes in inflammatory mediators and selected histopathological findings. These results suggest that NAC may attenuate AST, albumin, certain inflammatory mediators, and tissue alterations associated with high-dose amikacin exposure. Further studies are needed to clarify the mechanisms involved and determine the potential clinical relevance of these findings.

## 1. Introduction

Aminoglycosides are among the most widely studied of bactericidal antibacterials and remain an integral part of current antimicrobial therapy despite the availability of newer drugs. Their value in clinical practice lies in their rapid killing of aerobic Gram-negative bacilli while also retaining activity against certain Gram-positive species, mainly *Staphylococcus aureus* [1]. These properties make aminoglycosides important in situations where resistance to β-lactams and fluoroquinolones limits therapeutic options. In neonatal and pediatric practice, aminoglycosides continue to be eminent for empiric therapy of life-threatening infections. Gentamicin and amikacin are the treatments of choice for neonatal meningitis and sepsis, illnesses in which early empiric therapy can help with survival [2]. Clinical practice guidelines also recommend their use in severe pneumonia, peritonitis, biliary tract infection, and endocarditis, speaking to their widespread therapeutic application in children [3]. Evidence from high-burden settings supports such dependence: a multicenter point-prevalence study of Indian neonatal intensive care units reported more than half of hospitalized neonates to be on antimicrobials, with the single most prescribed antibiotic being amikacin [4]. This pattern highlights an essential paradox: aminoglycosides are among the safest and most accessible options for susceptible children, yet their ongoing overuse fuels growing concerns about toxicity.

The general need for these medications necessitates a commensurate evaluation of their worth relative to their potential for toxicity. While aminoglycosides have proven indispensable in many clinical conditions, their use is anything but risk-free: aminoglycoside therapy is notoriously limited by dose-dependent organ toxicities affecting multiple systems. Nephrotoxicity occurs in a high percentage of patients, typically manifesting as acute tubular necrosis and renal insufficiency [5,6]. Hepatotoxic effects are also well documented, evidenced by elevations in liver enzymes such as alanine aminotransferase (ALT), aspartate aminotransferase (AST), and alkaline phosphatase (ALP), along with markers of cholestatic injury [7,8,9]. These organ toxicities are severe and often irreversible, and hence, fundamentally constrain aminoglycoside safety [9]. On the molecular level, renal tissue damage caused by aminoglycosides is mediated in part by inflammatory markers modulations [10]; specifically, upregulated inflammatory cytokines were observed in animals exposed to amikacin. Amikacin exposure was associated with marked renal histopathological changes, including tubular and glomerular injuries, thinning of the tubular epithelium, and altered Bowman’s space [11].

Additionally, aminoglycosides induce inflammatory reactions, as damage to kidney, liver, and brain tissues causes the release of inflammatory substances and activates immune signals. Specifically, animals with aminoglycoside nephrotoxicity exhibit increased levels of TNF-α and IL-6 [12]. The drugs may also activate NF-κB pathways in the impacted tissues [12], starting a chain reaction of cytokine and chemokine production. This increase in inflammation is associated with renal tissue injury that accelerates kidney damage, as reported previously with cisplatin [13]. That is, immunological and inflammatory responses are correlated with each other: Amikacin may trigger inflammation, and the immune cells that arrive at the injury site stimulate cytokines and chemokines production, causing tissue damage. Correspondingly, understanding both inflammation and immune systems is key to protecting organs during aminoglycoside therapy [14].

One promising approach to protecting against aminoglycoside-induced oxidative damage is the co-administration of antioxidants [15]. *N*-acetylcysteine (NAC) is a potent thiol antioxidant and glutathione (GSH) precursor that has demonstrated cytoprotective activity in a number of organ systems [16,17], and its administration increases intracellular GSH, a key endogenous antioxidant [18]. GSH depletion is known to exacerbate oxidative damage; in fact, low GSH is associated with many pathological disorders involving oxidative stress, such as chronic renal failure and Parkinson’s disease [19]. The neuroprotective effects of NAC are also well demonstrated; for instance, in a mouse model of Alzheimer’s disease, NAC administration reduces ethanol-induced neuroinflmmatory mediators, an effect associated with improved cognitive behaviors [20]. Importantly, NAC is not an antimicrobial and does not inhibit the bactericidal activity of aminoglycosides, making it a supportive adjunct therapy with the specific utility of counteracting aminoglycoside-induced toxicity [21,22,23]. NAC is widely employed in clinical use for acetaminophen poisoning and as a mucolytic, and established as safe and well tolerated; it is thus highly amenable to repurposing as a protective agent in antibiotic treatment protocols.

Beyond TNF-α and IL-6 signaling, recent data indicate the inflammatory response in aminoglycoside toxicity to also involve other pathways. To examine this more closely and determine which inflammatory pathways are the most engaged, we assayed a broad panel of 20 inflammatory factors representing a variety of immune signaling pathways. We further conducted a preclinical study to investigate whether co-treatment with NAC could attenuate against amikacin-increased inflammatory markers in vivo. Specifically, we employed acute high-dose amikacin treatment in mice to mimic intensive amikacin therapy and its accompanying alterations, and examined the effects of NAC co-treatment on biochemistry profile, inflammatory markers, and selected histopathology markers. The findings of this study help illuminate the roles of anti-inflammatory mechanisms in aminoglycoside side effects and the potential of NAC as a therapeutic adjuvant to reduce the side effects of aminoglycosides.

## 2. Materials and Methods

### 2.1. Animal Model and Study Design

Male Swiss albino mice (at 6–8 weeks of age and an average body weight of 25–30 g) were housed and kept at a 12 h light/12 h dark cycle and provided ad libitum access to standard chow and water. Mice were obtained from the experimental animal care center, College of Pharmacy, King Saud University. All procedures complied with institutional animal care guidelines and were approved by the King Saud University Animal Ethics Committee (KSU-SE-25-34, approval date: 29 June 2025). Throughout the experimental period, the animals were monitored regularly for mortality and adverse clinical events such as pain, distress, or abnormal behavior. No mortality or apparent adverse clinical events were observed in any of the experimental groups during the study period; therefore, analgesic or other therapeutic interventions were not required.

### 2.2. Experimental Design

A total of 27 male Swiss albino mice were used and randomly allocated into four groups (*n* = 7/group). Mice were divided into four groups: group (1)—control group; group; (2)—amikacin group treated with 500 mg/kg s.c. for 5 days [24,25]; group (3)—amikacin/NAC group treated with amikacin (500 mg/kg s.c.) for 5 days and NAC (500 mg/kg i.p.) for 5 days [24,25,26]; and group (4)—NAC alone group treated with 500 mg/kg i.p for 5 days [26]. NAC was administered 1 h before s.c. injection of amikacin during the five days [24], indicating that the present study primarily evaluated a prophylactic approach. This approach may be relevant considering the role of NAC as a glutathione precursor and the dietary availability of cysteine, which supports the antioxidant defenses. Animals were fasted for 10 h before blood collection, with free access to water. The doses of amikacin and NAC used in this study were selected based on previous experimental studies. Amikacin at 500 mg/kg has been used in rodent models to induce nephrotoxicity and ototoxicity [24,25], while NAC at 500 mg/kg has demonstrated protective effects in preclinical models [24]. These doses were chosen to establish a reproducible experimental model that would permit the assessment of biochemical, inflammatory, and anxiety-like behavior. Body weight measurements and behavioral assessments were performed using all included animals. Serum biochemical and multiplex cytokine/chemokine analyses were conducted using samples from randomly selected five animals per group. The five animals were randomly selected from the seven animals in each experimental group. Each animal was assigned an identification number, and five animals were selected without consideration of their behavioral performance, body weight, water intake, or treatment response. The use of five animals per group for these analyses was based on the availability of sufficient serum volume for the planned biochemical assays and the sample capacity of the multiplex assay kit (Thermo Fisher Scientific, Waltham, MA, USA).

### 2.3. Behavioral Assessment

Behavioral performance was assessed using the light-dark box test. The test was performed on day 5 after the completion of the treatments. The test was conducted for 5 min per mouse 1 h after amikacin treatments. The light-dark box apparatus consisted of a rectangular Plexiglas box (45 cm × 27 cm × 27 cm) divided into two equally sized compartments by a partition with a small opening. For the light-dark box test, each mouse was placed in the center of the apparatus, facing the light compartment, and was allowed to freely explore both compartments for 5 min. The dark compartment was maintained under low illumination (<10 lux), while the light compartment was illuminated. The time spent in the light compartment was recorded as a measure of behavioral performance. The behavioral test was recorded using an overhead camera. Video data were analyzed using ANY-maze (version 6) software to automatically track mouse movements and calculate spatial parameters. Behavioral and body weight data analyses were performed by investigators blinded to group allocation.

### 2.4. Sample Collection and Biochemical Analyses

After completion of the treatment protocol, mice were anesthetized with isoflurane, and blood samples were collected via cardiac puncture under deep anesthesia. The animals were subsequently euthanized by decapitation. Blood samples were allowed to clot at room temperature for 30 min and centrifuged at 3000× *g* for 10 min to separate serum. Serum aliquots were stored at −80 °C for biochemistry and inflammatory analysis. Serum electrolytes, liver and kidney functions, serum proteins, lipid markers, lactate dehydrogenase (LDH), glucose, and others were measured using a biochemistry analytical machine (SCITEK, Jinan City, Shandong Province, China). The machine ran the samples for many parameters after loading 100 µL of serum onto a special disc. The sample processing was performed by researchers blinded to group allocation.

### 2.5. Inflammatory Marker Assessment

To characterize the inflammatory response in peripheral organs, a comprehensive panel of 20 cytokines and chemokines was quantified in serum using a customized multiplex bead-based immunoassay platform from Invitrogen (Carlsbad, CA, USA). Serum samples (25 μL per assay) were analyzed in duplicate following overnight incubation, as per the manufacturer’s instructions. Serum samples per well were quantified based on mean fluorescent intensity using Luminex FLEXMAP 3D system and xPONENT software v4.2 (Luminex Corporation, Austin, TX, USA). The results were calculated using a five-parameter logistic regression of the standard curve, processed with Belysa software v1.1.0 (Merck KGaA). The panel quantified 20 inflammatory mediators distributed across three bead mixes: Bead Mix B (8 analytes): GM-CSF, IL-2, IL-12p70, IL-13, IL-18, IL-5, IL-6, TNF-α; Bead Mix C (4 analytes): IL-17A, IL-22, IL-23, IL-27; Bead Mix D (8 analytes): CCL11 (Eotaxin), CXCL1 (KC), CXCL10 (IP-10), CCL2 (MCP-1), CCL7 (MCP-3), CCL3 (MIP-1α), CCL5 (RANTES), and CXCL2 (MIP-2α). The sample processing was performed by researchers blinded to group allocation. Measurements that were below the assay detection limit or could not be reliably quantified by the instrument were excluded from the corresponding cytokine analysis.

### 2.6. Histopathological Assessment

Brains and kidneys were extracted, preserved in 4% formaldehyde immediately and then paraffin-embedded. A microtome was used for renal and cerebral cortex sectioning; the sections were then stained with hematoxylin and eosin (H&E). To assess the extent of renal and brain injury, the cellular structure, renal glomerulus, renal Bowman’s space, brain degeneration, and gliosis-like changes were examined using an optical microscope (Olympus BX microscope and DP72 camera, Melville, FL, USA). Inflammatory cell infiltration was assessed according to the extent and distribution of inflammatory cells within the examined cortical fields. Histopathological evaluation was performed on tissue samples from five animals per group by an experienced histopathologist blinded to the treatment groups. For renal histopathology, two randomly selected non-overlapping cortical fields were examined from each animal, and the histopathological scores from the two fields were averaged to generate a single score for that animal (×100 magnification) according to the following criteria: glomerular atrophy, interstitial inflammation, tubular cast, tubular degeneration, and tubular dilatation [27]. It was scored as 0 = no injury, 1 = mid less than 25% injury, 2 = moderate 25–50% injury, and 3 = severe, more than 50% injury. For cerebral histopathology, five randomly selected non-overlapping cortical fields were examined per animal, and the histopathological scores were assigned based on the overall assessment of these fields (×100 magnification) [28]. A semi-quantitative pathological scoring system for the cerebral cortex typically evaluated neuronal necrosis, edema, hemorrhage, and gliosis-like changes (0 = non; 1 = mild; 2 = moderate; 3 = severe). Statistical analyses were performed using the individual animal as the experimental unit.

### 2.7. Statistical Analysis

The obtained data were statistically analyzed using GraphPad Prism (version 6.01) software. Two-way ANOVA was used to track and examine the average body weight and water intake readings. Pairwise comparisons for behavioral, molecular, and histopathological data were performed using Fisher’s protected least significant difference (LSD) test only when the overall one-way ANOVA was significant. No outliers were excluded from the analyses, and no missing data were present in the dataset. Statistical significance was fixed at *p* < 0.05. To account for multiple testing in the cytokines and chemokines data analyses, the resulting *p*-values were additionally adjusted using the Benjamini–Hochberg false discovery rate (FDR) procedure. Analytes with an FDR-adjusted *p*-value of <0.05 were considered statistically significant following correction for multiple comparisons (Supplementary Table S1).

### 2.8. Heatmap and Spls-DA Analysis of Cytokine Profiles, and the Overall Data Summary

MetaboAnalystR software (version 6.0) was used to generate Sparse Partial Least Squares Discriminant Analysis (Spls-DA), parameters correlations, and heatmap analysis for biochemistry and inflammatory profiles of the studied groups. Biochemistry and inflammatory data were normalized to the average of control group data. For Spls-DA, missing values were filtered using a 20% missing-value threshold. The data were then normalized using the default MetaboAnalystR normalization workflow, including data centering and autoscaling (unit variance scaling), before model construction. The Spls-DA model was constructed using two latent components with 10 selected variables (keepX) per component. The number of latent components was selected based on cross-validation performance to minimize classification error while avoiding overfitting. Model performance was evaluated using repeated 5-fold M-fold cross-validation, and classification performance was assessed using the cross-validation module implemented in MetaboAnalystR. Model robustness was further evaluated using PLS-DA-based permutation test with 1000 permutations. Statistical significance of the multivariate group separation was additionally assessed using PCA score-based permutational multivariate analysis of variance (PERMANOVA) with 999 permutations. OpenAI’s ChatGPT image generation tool (GPT-5.5; OpenAI, San Francisco, CA, USA) was used solely to generate graphical illustrations summarizing the study design, the experimental findings and the future directions.

## 3. Results

### 3.1. Effects of Treatment on Body Weight and Water Intake

**Figure 1:**
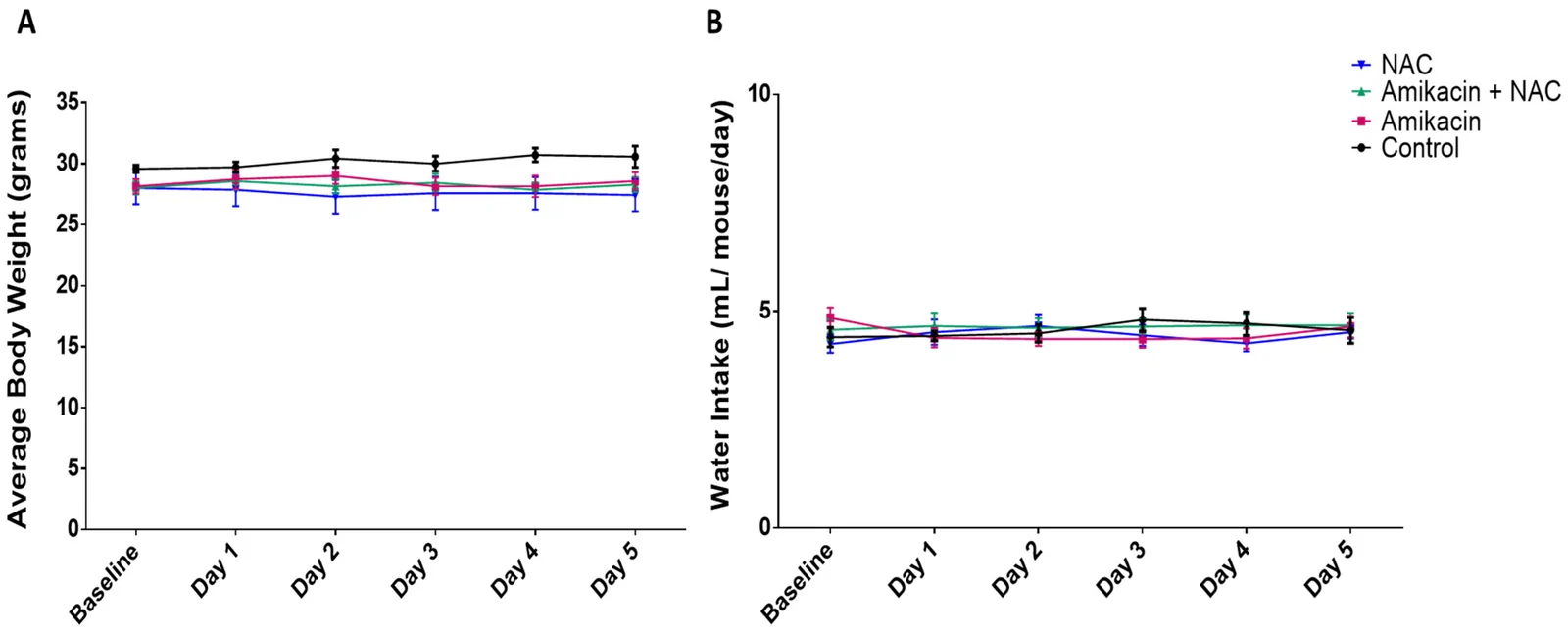
Effects of treatment on average body weight and water intake. (**A**) The line graph displays the change in average body weight (in grams) for all four treatment groups over the 5-day experimental period. (**B**) The line graph displays the change in water intake (in Ml/mouse/day) for all four treatment groups over the 5-day experimental period. Two-way ANOVA revealed non-significant differences between the studied groups throughout the experiment. Data are presented as mean ± SEM (*n* = 7/group).

The effects of the treatments were monitored on the body weights and water intake by tracking average body weight and water consumptions over the 5-day experimental period (Figure 1A,B). It showed no significant differences in average body weight or water consumption between the control and treatment groups (Control, Amikacin, NAC, Amikacin + NAC) at baseline or at any point during the 5-day study using two-way ANOVA.

### 3.2. Behavioral Analysis

#### 3.2.1. Assessment of Anxiety-like Behavior in the Light−Dark Box Test

**Figure 2:**
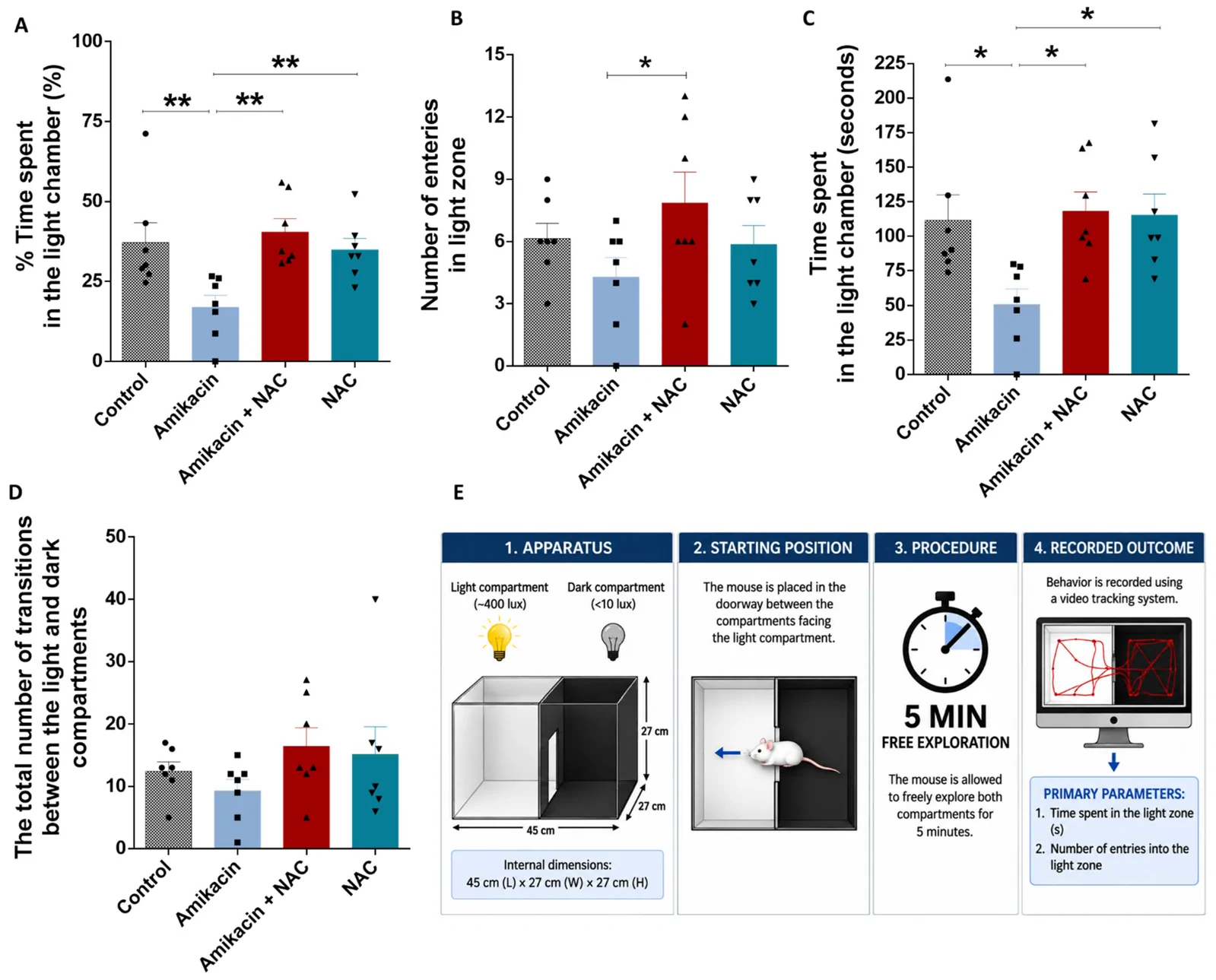
Quantitative analysis of behavioral performance in the light-dark box test. (**A**) The statistical analysis showed that amikacin has a significant lower percentage of time spent in the light chamber as compared to the other three groups (*p* < 0.01). (**B**) Although one-way ANOVA did not show differences in light zone entries among the groups, the statistical analysis showed that the total number of entries into the light zone was significantly higher in the amikacin-NAC group as compared to in the amikacin group (*p* < 0.05). (**C**) The statistical analysis showed that amikacin has significant lower total time spent in the light chamber as compared to the other three groups (*p* < 0.05). (**D**) The statistical analysis did not show any statistical alterations in the total number of transitions between the light and dark compartments between the groups. (**E**) Experimental setup and procedure of the light/dark box test. Data are presented as mean ± SEM (*n* = 7/group). * *p* < 0.05; ** *p* < 0.01.

The light-dark box test was conducted to determine if amikacin induced anxiety-like behavior (Figure 2). One-way ANOVA showed significant alterations in the percentage of time spent in the light chamber (F (3, 24) = 5.539, *p* = 0.0049) and the total time in seconds in the light chamber (F (3, 24) = 4.674, *p* = 0.0104) among the groups. The post-hoc analysis showed that the amikacin-treated group demonstrated significant anxiety behavior compared to the control group. This was evidenced by a marked reduction in the percentage of time spent in the light chamber (Figure 2A) and the total time in seconds in the light chamber (Figure 2C). Although one-way ANOVA did not show differences in light zone entries among the groups (F (3, 24) = 1.913, *p* = 0.1544), the statistical analysis showed that the total number of entries into the light zone was significantly higher in the amikacin-NAC group as compared to in the amikacin group (*p* < 0.05) (Figure 2B). These behavioral changes were effectively prevented by the co-administration of NAC. The amikacin-NAC group’s performance across all three quantitative measures was statistically indistinguishable from the control group, indicating a return to a normal behavioral phenotype.

#### 3.2.2. Biochemistry Profile: Comprehensive Biochemistry Response Analysis

**Figure 3:**
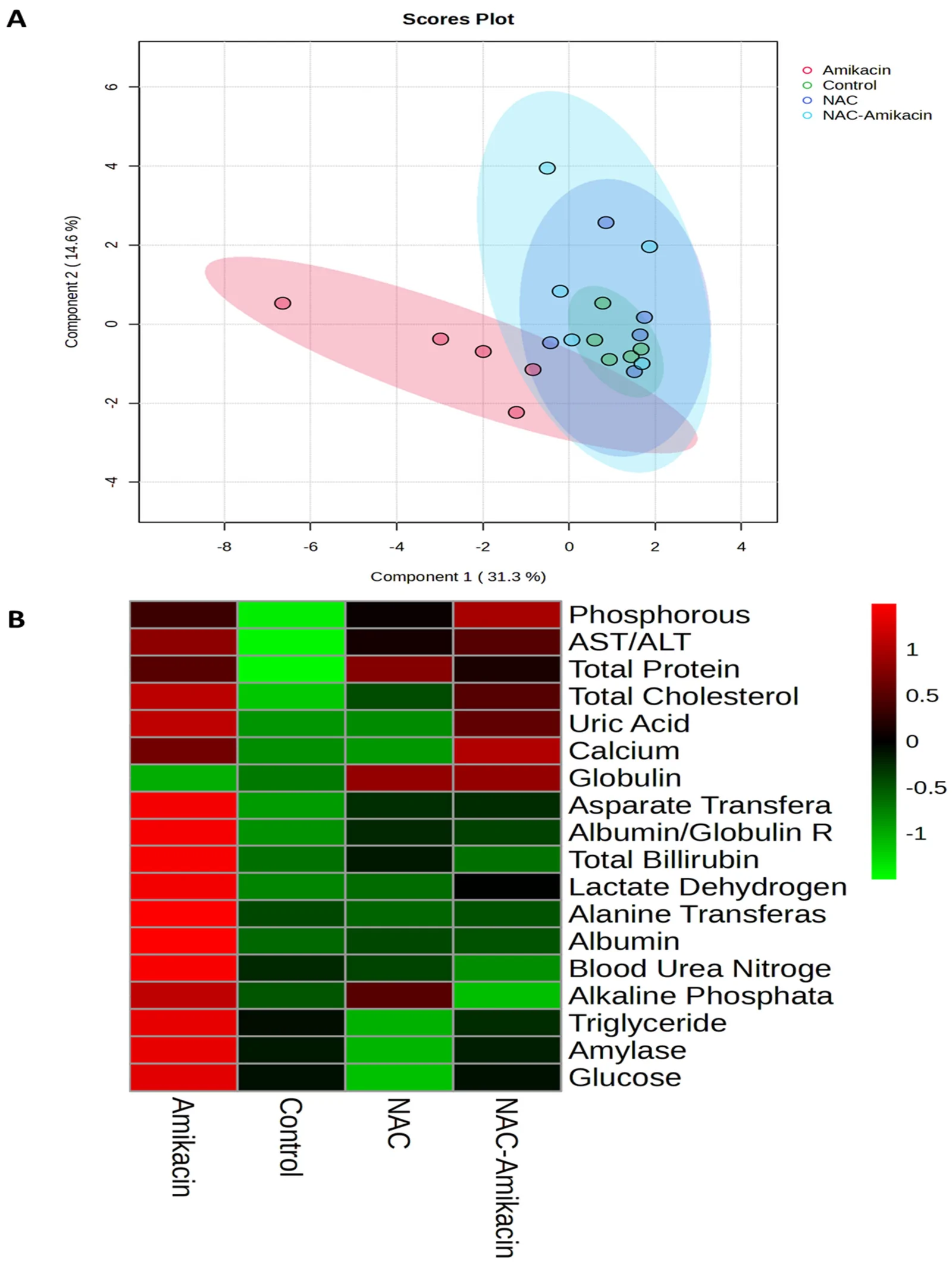
(**A**) Sparse partial least squares discriminant analysis (Spls-DA) of the biochemical profile. (**B**) Heatmap of the biochemical profile with Pearson correlations. Red cells indicate high concentration, while green cells indicate low concentration.

Spls-DA was performed to identify patterns of biochemical changes across treatment groups (Figure 3A). Exploratory PLS-DA of the biochemical profile showed limited separation among the experimental groups. However, permutation testing indicated that the observed separation was not statistically significant (permutation *p* = 0.132), suggesting that the discrimination could have occurred by chance. Consistent with this finding, PERMANOVA did not detect significant overall differences in the biochemical profiles among the four groups (F = 1.8093, *p* = 0.15; 199 permutations). Pairwise PERMANOVA identified a nominal difference between the amikacin and control groups before multiple-testing correction (raw *p* = 0.040), but this difference was no longer significant after adjustment (adjusted *p* = 0.210). No other pairwise comparisons reached statistical significance after correction (all adjusted *p* > 0.20). To visualize the relative changes in individual markers across the groups, a heatmap was generated (Figure 3B). This overview clearly demonstrates a distinct biochemical profile for the amikacin-treated group, characterized by a widespread increase (red) in markers of organ injury and metabolic stress. In contrast, the NAC–amikacin group showed a profile more similar to that of the control group, suggesting attenuation of the amikacin-associated alterations by NAC.

**Figure 4:**
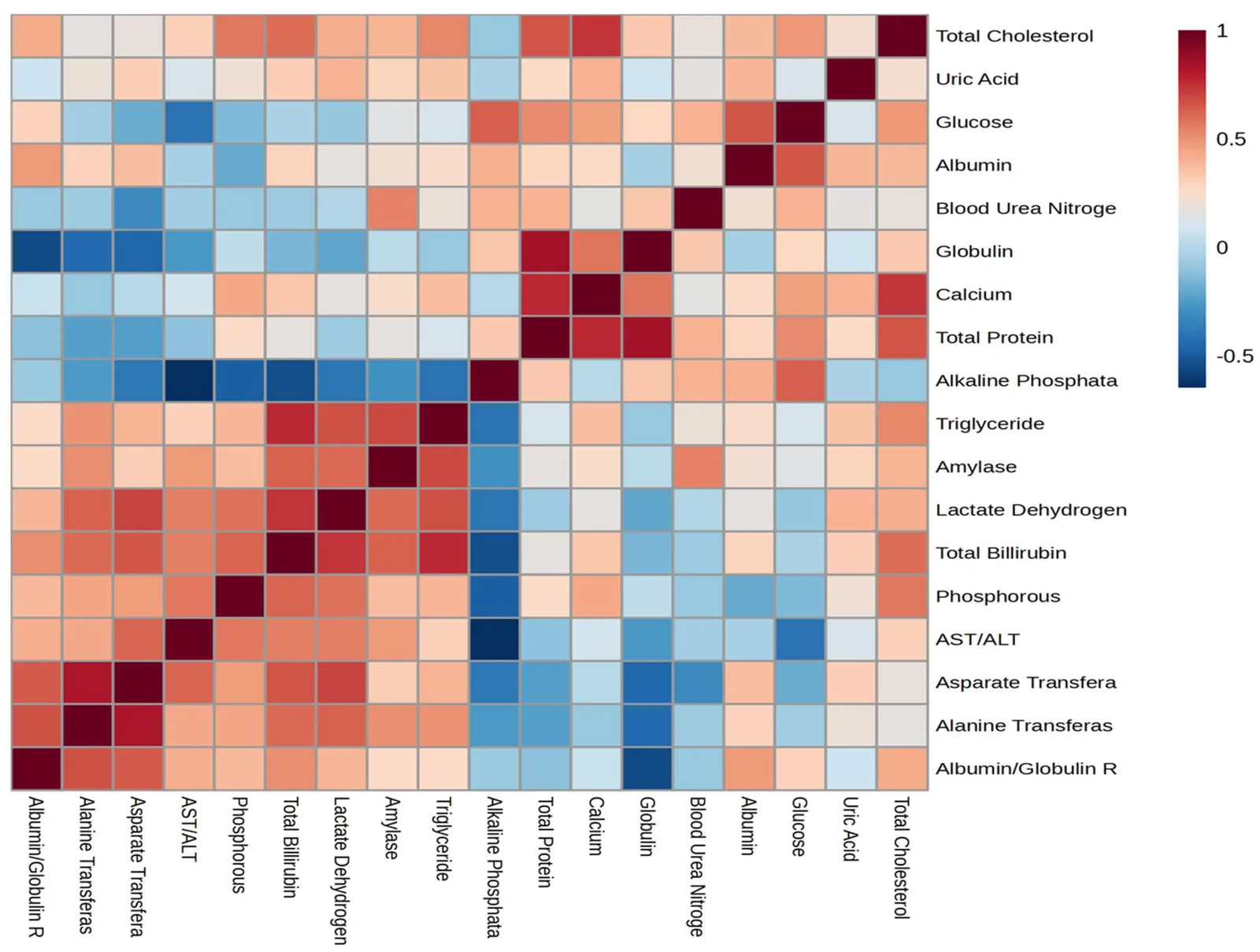
Biochemistry markers correlation. Red cells indicate positive correlations, while blue cells indicate negative correlations.

The correlation analysis revealed strong positive correlations between calcium and total protein, cholesterol and total protein, alkaline phosphatase and glucose, albumin and glucose, cholesterol and bilirubin, cholesterol and calcium, AST and AST/ALT, LDH and AST, LDH and ALT, LDH and amylase, LDH and triglyceride, LDH and bilirubin, ALT and bilirubin, AST and bilirubin, triglyceride and bilirubin, inorganic phosphate and bilirubin, triglyceride and amylase, ALT and albumin/globulin, and calcium/inorganic phosphate and AST (Figure 4). Very strong positive correlations were found in total protein and globulin, and AST and ALT. The analysis also revealed strong negative correlations between alkaline phosphatase and AST/ALT (Figure 4).

#### 3.2.3. Effects of Treatments on Selected Hepatic Function Tests

**Figure 5:**
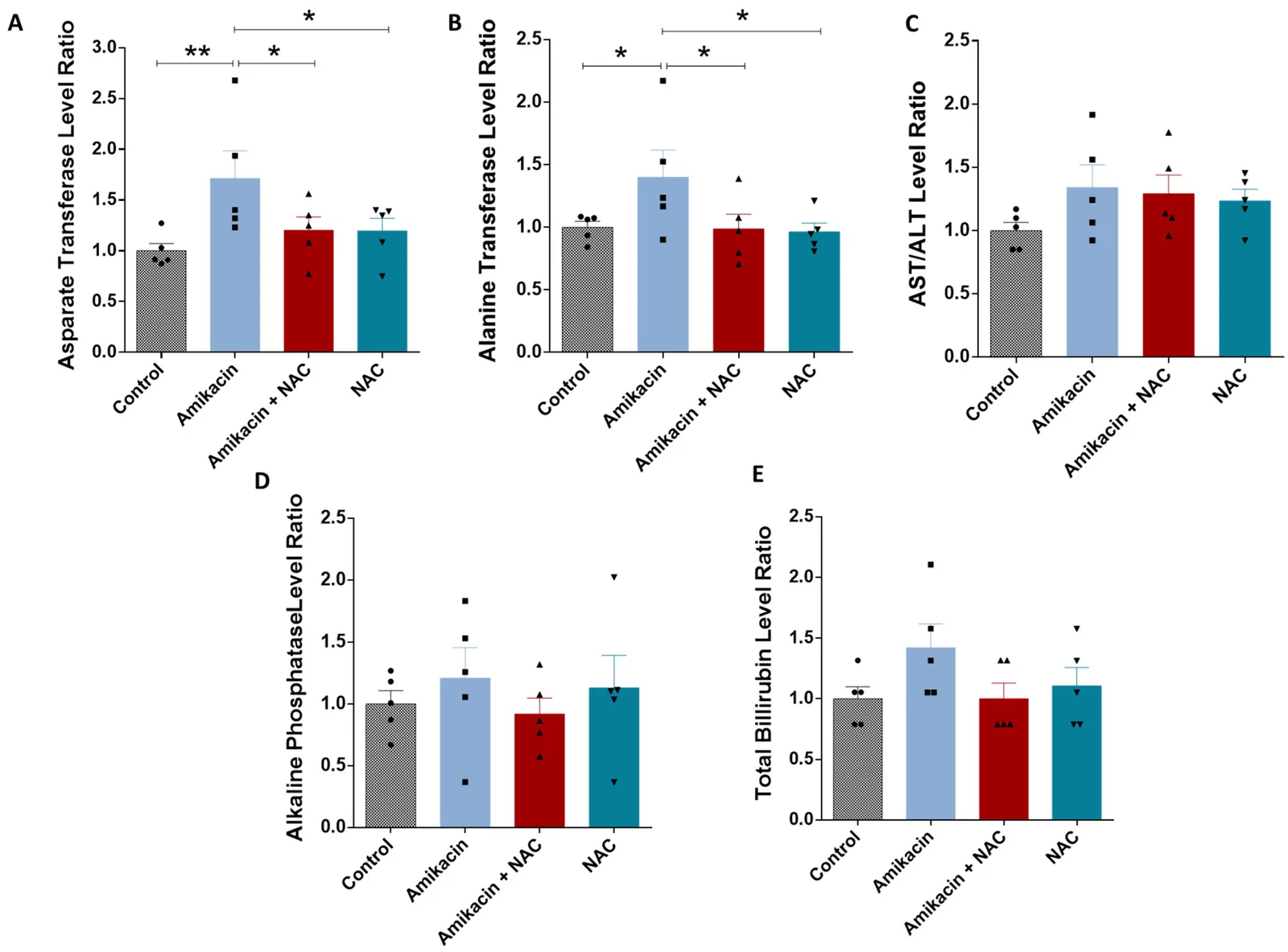
Assessment of liver function markers. (**A**) Aspartate aminotransferase (AST) levels was significantly increased in the amikacin group compared to in the other three groups. (**B**) Alanine aminotransferase (ALT) levels were significantly increased in the amikacin group as compared to the control (*p* < 0.01), amikacin-NAC (*p* < 0.05), and NAC (*p* < 0.05) groups. (**C**) The AST/ALT ratio was significantly increased in the amikacin group as compared to the control group. (**D**) Alkaline phosphatase was not altered between the four groups. (**E**) Total bilirubin was not altered between the four groups. Data are presented as mean ± SEM (*n* = 5/group). * *p* < 0.05; ** *p* < 0.01.

Comprehensive biochemical profiling revealed significant alterations in the liver function markers following amikacin treatments. One-way ANOVA and post-hoc analysis revealed that the liver function enzyme, aspartate transaminase (AST), was significantly in the amikacin group compared to in the other three groups (F (3, 16) = 3.333, *p* = 0.0461) (Figure 5A). Although one-way ANOVA showed no significant changes in alanine transaminase (ALT) levels among the groups (F (3, 16) = 2.555, *p* = 0.0917), the post-hoc revealed that ALT was increased in the amikacin group as compared to the other three groups (Figure 5B). The AST/ALT ratio was not changed among the four groups (Figure 5C). NAC treatment attenuated the amikacin-associated elevations in these transaminases, maintaining levels closer to control values. The statistical analysis did not reveal any significant changes in the alkaline phosphatase activity between the studied groups (Figure 5D). We also found that the total bilirubin level was not significantly changed between groups (Figure 5E).

#### 3.2.4. Effects of Treatments on Glucose, Blood Urea Nitrogen, Uric Acid, and LDH

**Figure 6:**
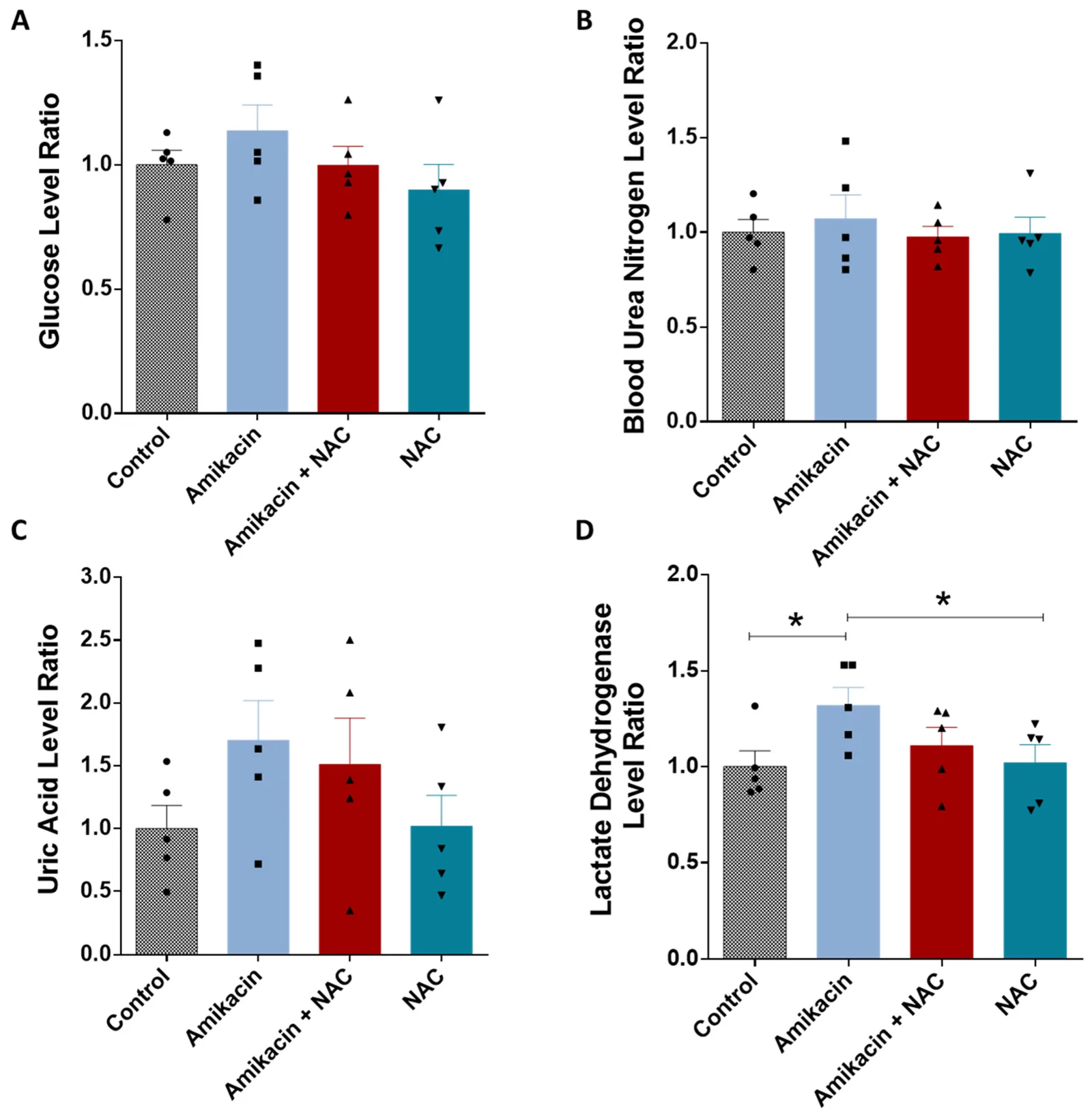
Assessment of glucose, blood urea nitrogen, uric acid, and lactate dehydrogenase. (**A**) The glucose level was not altered between the four groups. (**B**) The blood urea nitrogen level was not altered between the four groups. (**C**) The uric acid level was not altered between the four groups. (**D**) Lactate dehydrogenase was significantly increased in the amikacin group as compared to the control (*p* < 0.05) or amikacin-NAC (*p* < 0.05) groups. Data are presented as mean ± SEM (*n* = 5/group). * *p* < 0.05.

The statistical analysis did not reveal any significant alterations on glucose (Figure 6A) or blood urea nitrogen (Figure 6B) between the four groups. Our work showed that the uric acid level was not significantly changed between groups (Figure 6C). One-way ANOVA did not reveal any significant alterations in the LDH serum level among the four groups (F (3, 16) = 2.501, *p* = 0.0964); however, LDH was increased in amikacin as compared to the control and NAC groups (Figure 6D).

#### 3.2.5. Effects of Treatments on Serum Proteins

**Figure 7:**
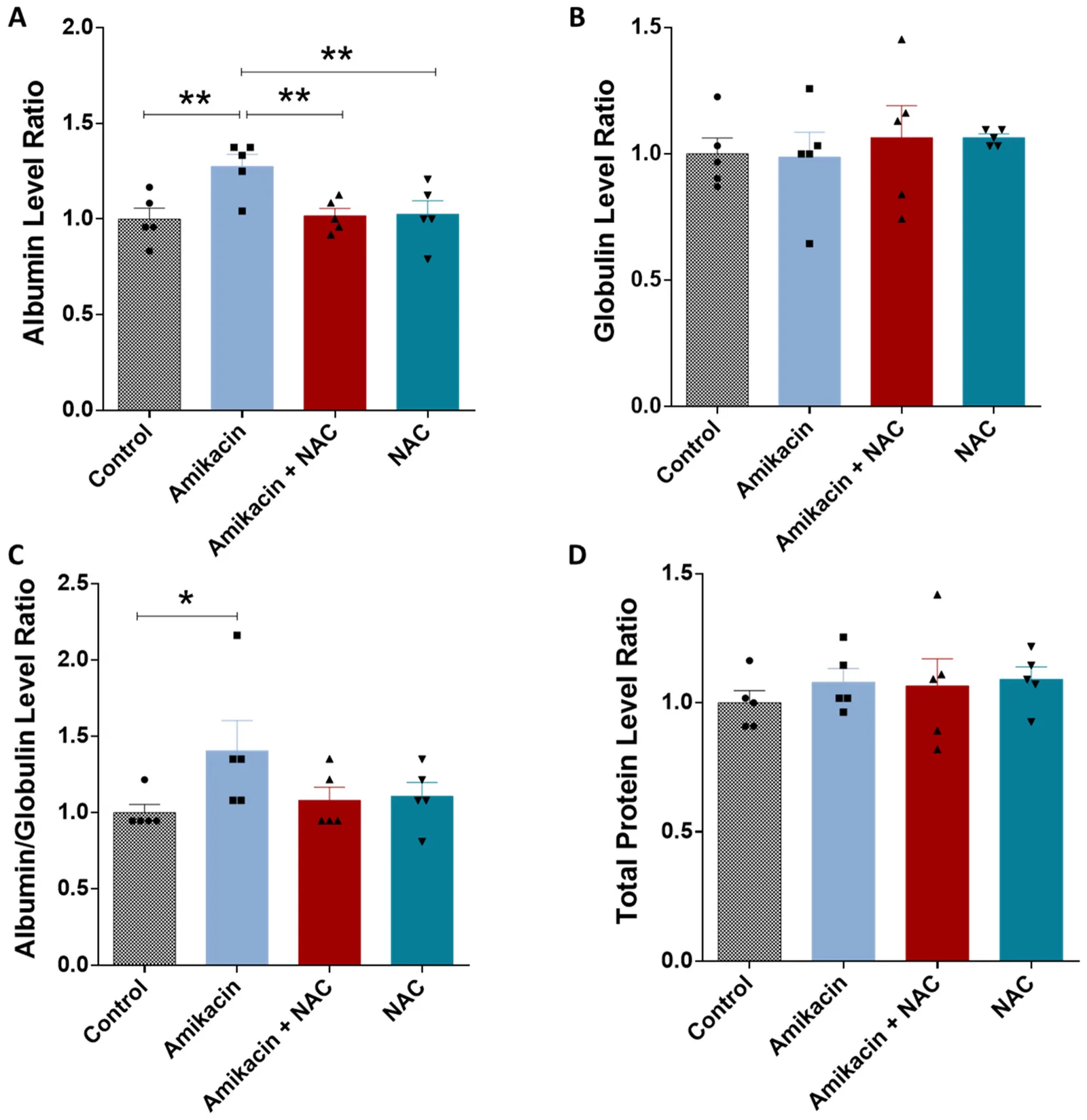
Assessment of serum proteins. (**A**) The albumin level was significantly higher in the amikacin group as compared to the other three groups (*p* < 0.01). (**B**) The globulin level was not changed between the four groups. (**C**) The albumin/globulin ratio was significantly higher in the amikacin group as compared to the control group (*p* < 0.05). (**D**) The total protein level was not changed between the four groups. Data are presented for each treatment group. Data are presented as mean ± SEM (*n* = 5/group). * *p* < 0.05; ** *p* < 0.01.

One-way ANOVA showed significant changes in albumin levels among the groups (F (3, 16) = 5.014, *p* = 0.0122). The post-hoc analysis revealed that amikacin treatment induced a significant increase in the serum albumin levels as compared to the other three groups (Figure 7A). Although the albumin/globulin ratio was higher in the amikacin group as compared to control group (Figure 7C), the analysis did not show statistical effects among the groups (F (3, 16) = 2.177, *p* = 0.1305). The statistical analysis did not show any significant changes on either globulin (Figure 7B) or total protein (Figure 7D) between the studied groups.

#### 3.2.6. Effects of Treatment on Lipid Markers, Calcium, and Inorganic Phosphate

**Figure 8:**
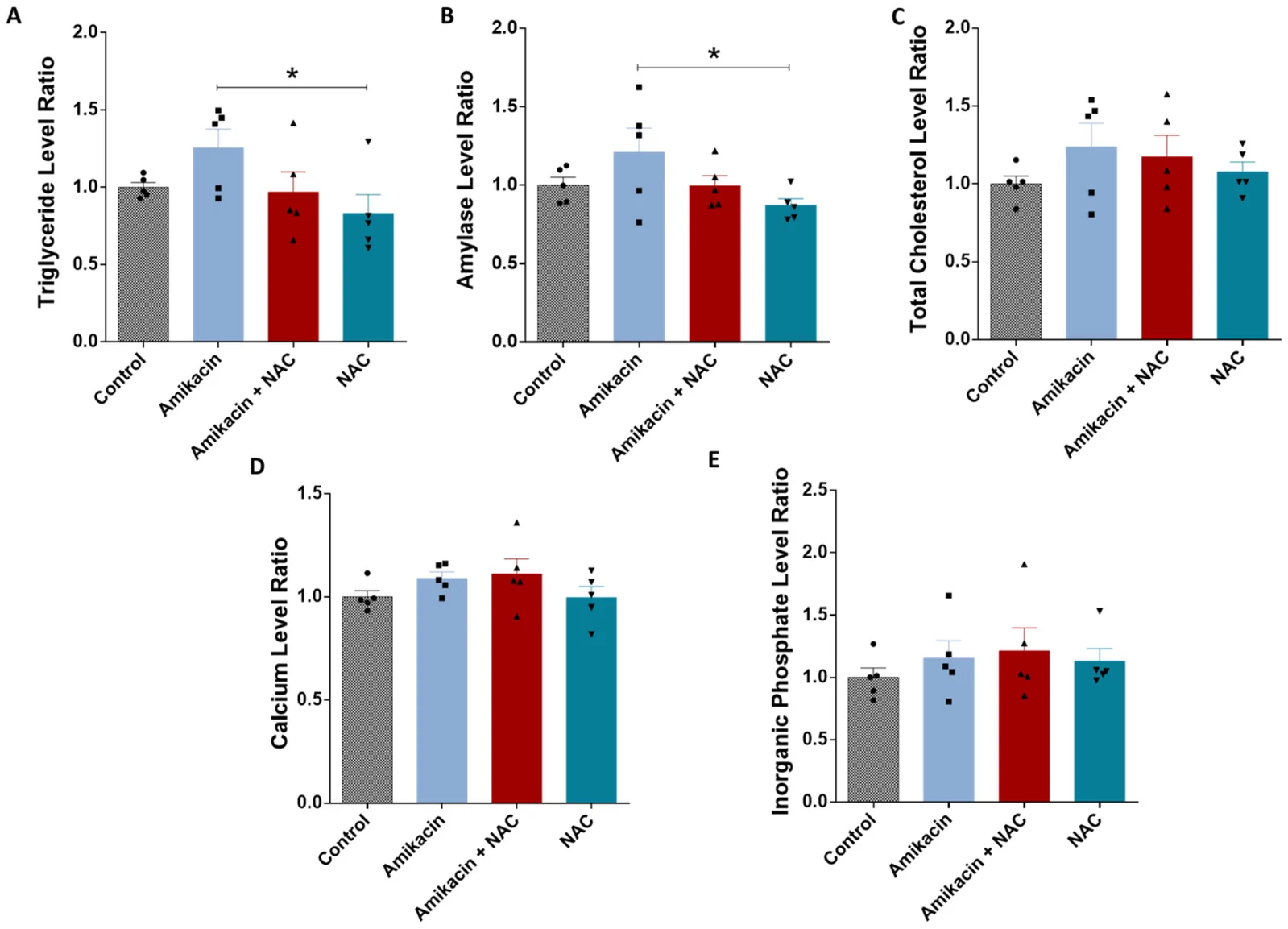
Assessment of lipid markers, calcium, and inorganic phosphate. (**A**) The triglyceride level was significantly higher in the amikacin group as compared to the NAC group (*p* < 0.05). (**B**) The amylase level was significantly higher in the amikacin group as compared to the NAC group (*p* < 0.05). (**C**) The total cholesterol level was not changed between the studied groups. (**D**) Calcium levels were not changed between the studied groups. (**E**) Inorganic phosphate levels were not changed between the studied groups. Data are presented for each treatment group. Data are presented as mean ± SEM (*n* = 5/group). * *p* < 0.05.

Although statistical analysis did not show alterations in serum triglyceride (F (3, 16) = 2.642, *p* = 0.0848) and amylase (F (3, 16) = 2.464, *p* = 0.0998) among the groups, serum levels were elevated in the amikacin group as compared to the NAC-only group (Figure 8A,B). However, the statistical analysis did not show any significant alterations in the serum cholesterol between the studied groups (Figure 8C). Statistical analysis did not show any significant changes on the serum calcium and inorganic phosphate concentrations between the four groups (Figure 8D,E).

#### 3.2.7. Inflammatory Profile: Comprehensive Inflammatory Response Analysis

**Figure 9:**
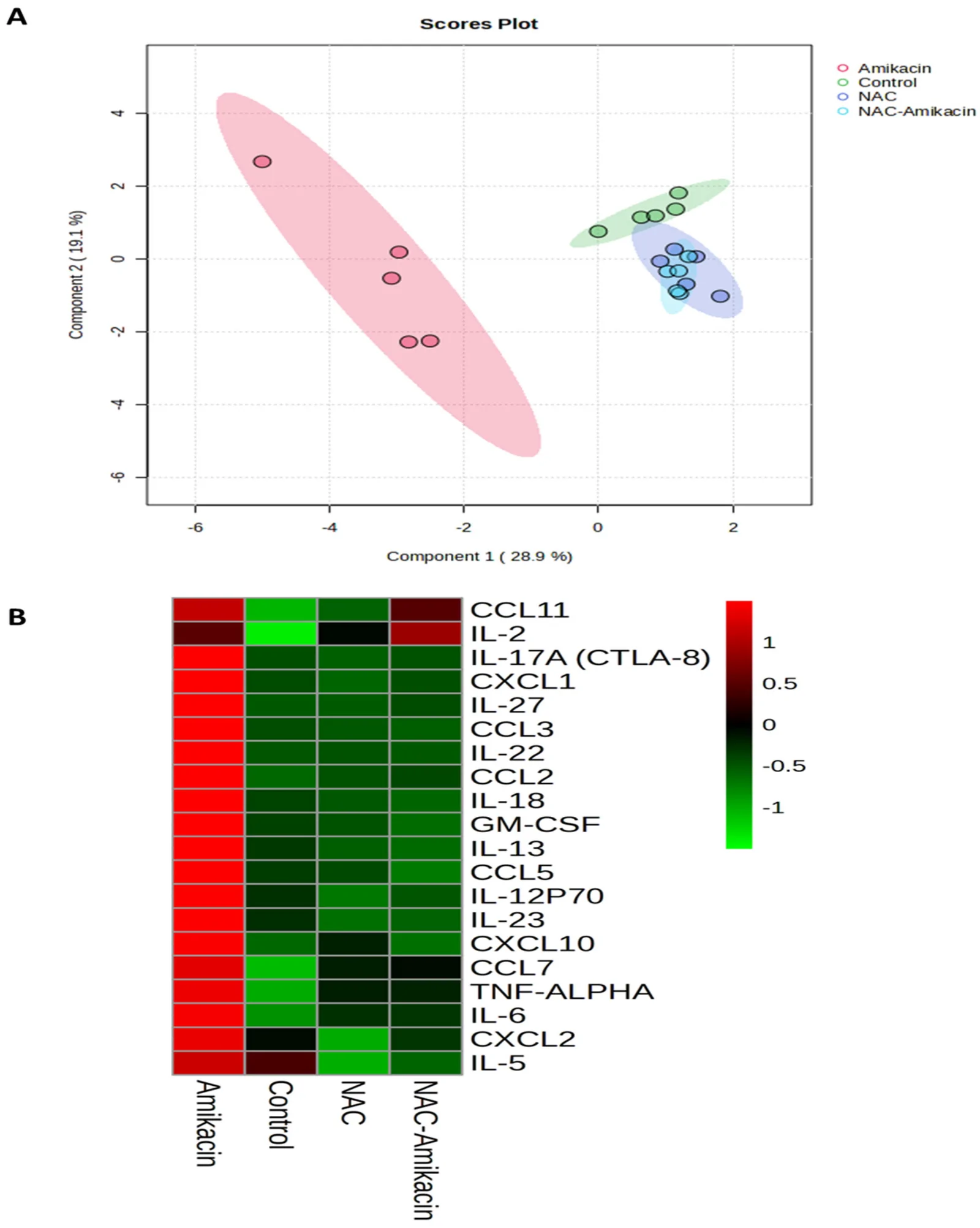
(**A**) Sparse partial least squares discriminant analysis (Spls-DA) of the inflammatory profile. (**B**) Heatmap of the inflammatory profile with Pearson correlations. Red cells indicate high concentration, while green cells indicate low concentration.

To assess the systemic inflammatory responses, a panel of cytokines and chemokines was analyzed. Spls-DA of the inflammatory profiles revealed a stark separation of the amikacin-treated group from the other groups (Figure 9A). Importantly, Spls-DA demonstrated separation among the four experimental groups. The first two latent components explained 28.9% and 19.1% of the total variance, respectively, accounting for 48.0% of the cumulative variance. Model robustness was evaluated using a permutation test with 1000 permutations. The observed model statistic was significantly greater than those obtained from randomly permuted datasets (permutation *p* = 0.005), indicating that the observed group separation was unlikely to have occurred by chance. In addition, PERMANOVA analysis confirmed a significant overall difference in cytokine profiles among the four experimental groups (F = 2.9234, *p* = 0.002; 999 permutations), indicating that the discrimination among groups was unlikely to have occurred by chance. Variables contributing most strongly to group separation included IL-12p70, IL-18, IL-22, IL-5, IL-6, CXCL1, CXCL10, IL-17A, and CCL11. Pairwise comparisons revealed that the amikacin group differed significantly from the control (adjusted *p* < 0.05), NAC (adjusted *p* < 0.05), and NAC + amikacin (adjusted *p* < 0.05) groups. In contrast, no significant differences were observed between the control, NAC, and NAC + amikacin groups after adjustment for multiple comparisons (all adjusted *p* > 0.80). Cross-validation demonstrated that the classification error increased with the number of latent components. The lowest error rate (35%) was observed for the one-component model, while the two-component model yielded a comparable error rate (40%) and was selected because it provided improved visualization of group separation. Additional components increased the classification errors. The control, NAC, and NAC−amikacin groups clustered tightly together, suggesting that NAC co-administration was associated with attenuation of the inflammatory profile induced by high-dose amikacin. To visualize the expression of individual mediators, a heatmap was generated (Figure 9B). This overview clearly demonstrates widespread elevations (red) of nearly all measured cytokines and chemokines in the amikacin group. In contrast, the NAC−amikacin and control groups show a profile of low levels (green), suggesting attenuation of some inflammatory mediators following NAC co-administration.

**Figure 10:**
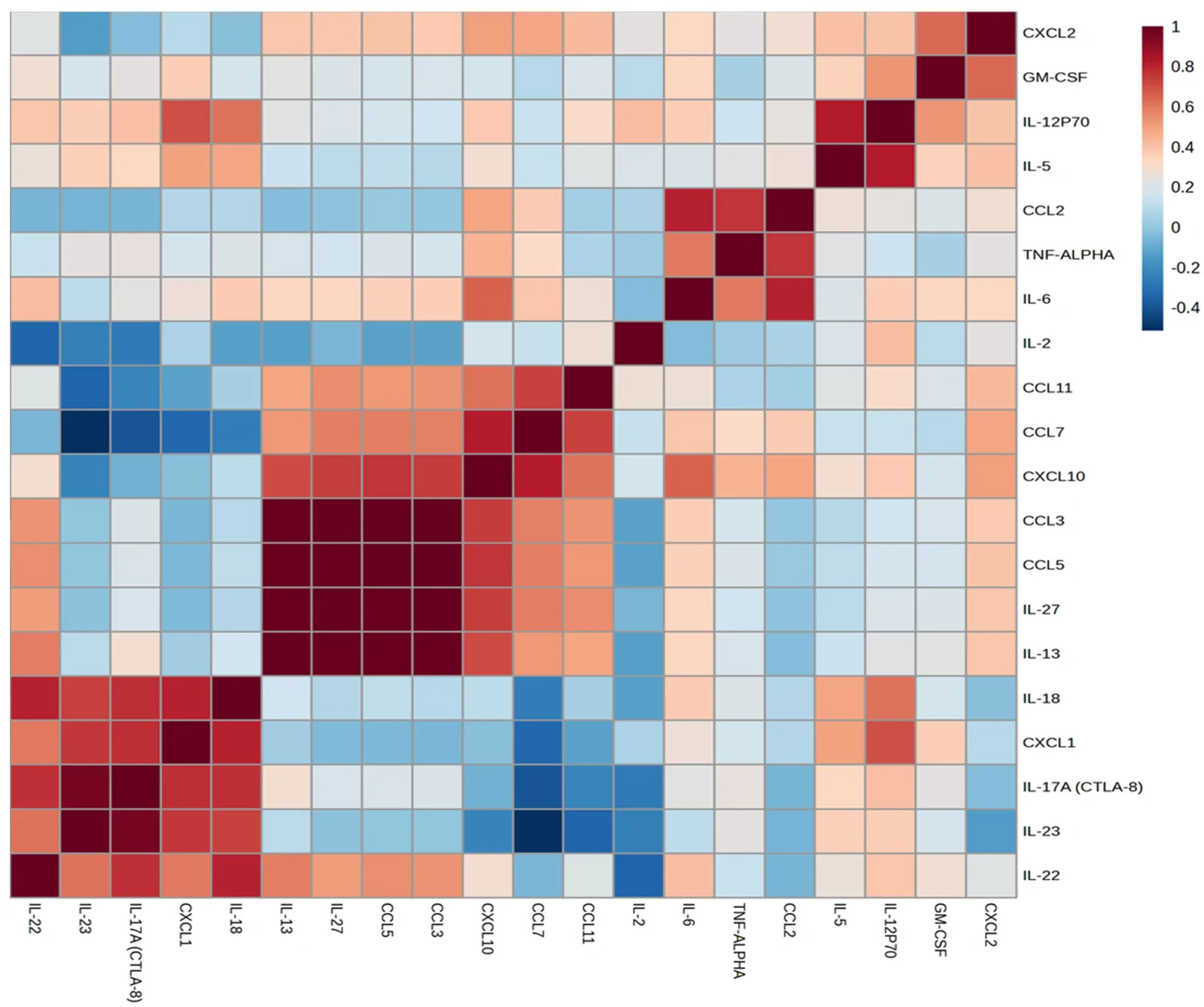
Correlation of cytokines and chemokines markers. Red cells indicate positive correlations, while blue cells indicate negative correlations.

The correlation analysis revealed very strong positive correlations between IL-17A and IL-23, IL-18 and IL-22, IL-18 and CXCL1, IL-13 and IL-27, IL-13 and CCL5, IL-27 and CCL5, IL-13 and CCL3, IL-27 and CCL3, CCL5 and CCL3, CCL7 and CXCL10, IL-6 and CCL2, and IL-12P70 and IL-5 (Figure 10). Moreover, strong positive correlations were found in IL-23 and IL-22, IL-17A and IL-22, CXCL1 and IL-22, CXCL1 and IL-23, CXCL1 and IL-17A, IL-18 and IL-23, IL-18 and IL-17A, CXCL10 and IL-13, CXCL10 and IL27, CXCL10 and CCL5, CXCL10 and CCL3, CCL11 and CXCL10, CCL11 and CCL7, CXCL10 and IL-6, TNF-α and CCL2, IL-12P70 and CXCL1, IL-12P70 and IL-18, and GM-CSF and CXCL2 (Figure 10).

#### 3.2.8. Effects of Treatments on Key Pro-Inflammatory Cytokines

**Figure 11:**
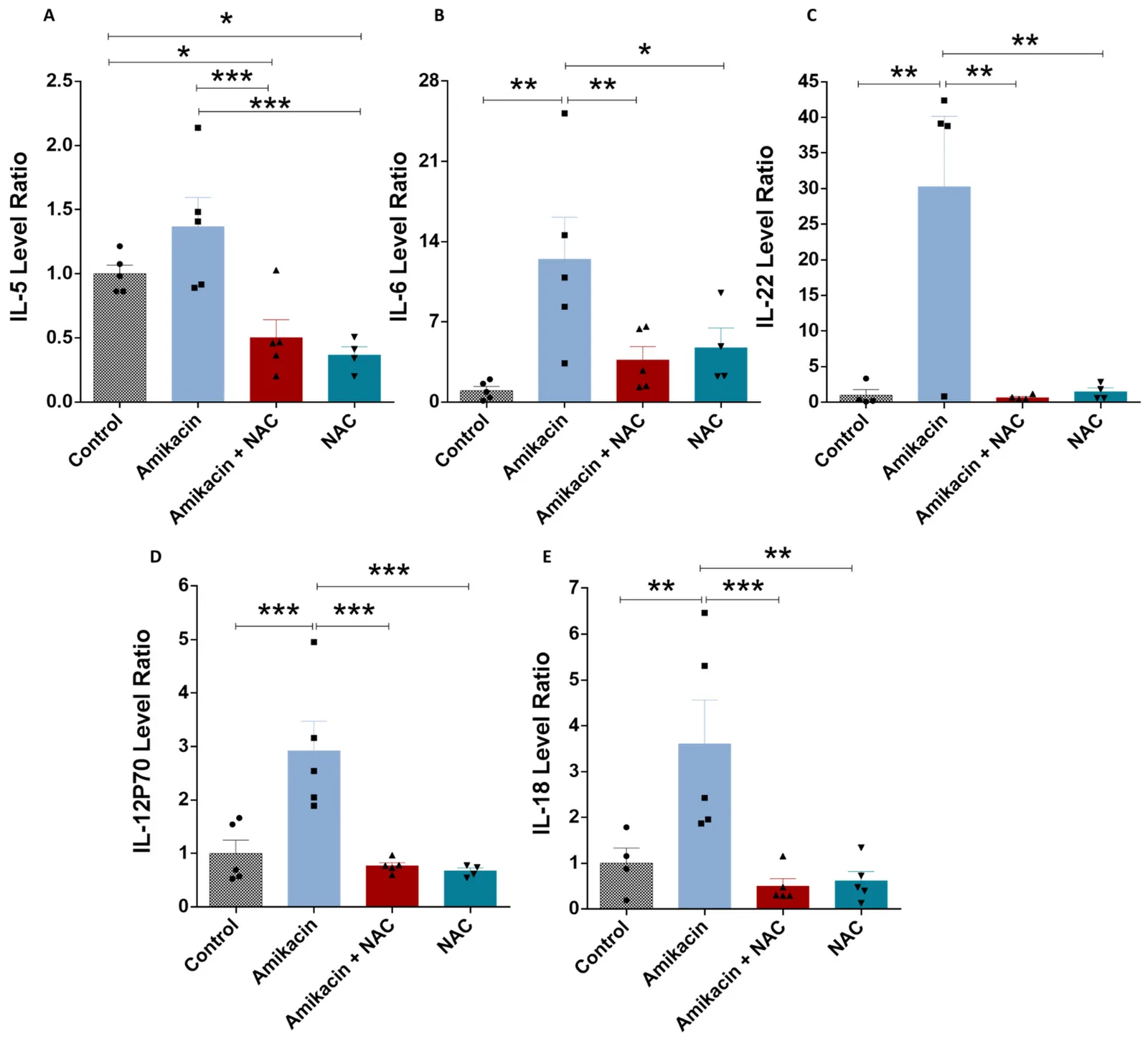
Assessment of pro-inflammatory cytokines. (**A**) The IL-5 level was significantly reduced in the amikacin-NAC group as compared to the control (*p* < 0.05) and amikacin (*p* < 0.001) groups, and it was also significantly decreased in the NAC group as compared to the control (*p* < 0.05) or amikacin groups (*p* < 0.001). (**B**) The IL-6 level was significantly higher in the amikacin group as compared to the control (*p* < 0.01), amikacin-NAC (*p* < 0.01), and NAC (*p* < 0.05) groups. (**C**) The IL-22 level was significantly higher in the amikacin group as compared to the other three groups (*p* < 0.01). (**D**) The IL-12P70 level was significantly higher in the amikacin group as compared to in the other three groups (*p* < 0.001). (**E**) The IL-18 level was significantly higher in the amikacin group as compared to the control (*p* < 0.01), amikacin-NAC (*p* < 0.001), or NAC (*p* < 0.01) groups. Data are presented for each treatment group. Data are presented as mean ± SEM (*n* = 4–5/group). * *p* < 0.05; ** *p* < 0.01; *** *p* < 0.001.

One-way ANOVA revealed significant alterations in the serum of interleukin-5 (IL-5) (F (3, 15) = 9.516, *p* = 0.0009), IL-6 (F (3, 15) = 5.441, *p* = 0.0098), IL22 (F (3, 12) = 8.725, *p* = 0.0024), IL-12P70 (F (3, 15) = 10.98, *p* = 0.0005), and IL-18 (F (3, 15) = 7.654, *p* = 0.0025) among the four studied groups. Amikacin treatment led to a marked elevation in several key pro-inflammatory cytokines, as detailed in Figure 11. IL-6, a central mediator of the acute phase response, was significantly elevated in the amikacin group as compared to the control group. In addition, IL-18, IL-5, IL-22, and IL-12P70, cytokines crucial for inflammatory responses, were also markedly increased in the amikacin group as compared to the controls. IL-5, a critical cytokine for eosinophil activity regulation, was increased in NAC-treated groups as compared to controls or amikacin. Co-treatment with NAC reversed the amikacin-induced increases in the levels of IL-5, IL-6, IL-22, IL-18, and IL-12P70.

#### 3.2.9. Effects of Treatments on Key Chemokines

**Figure 12:**
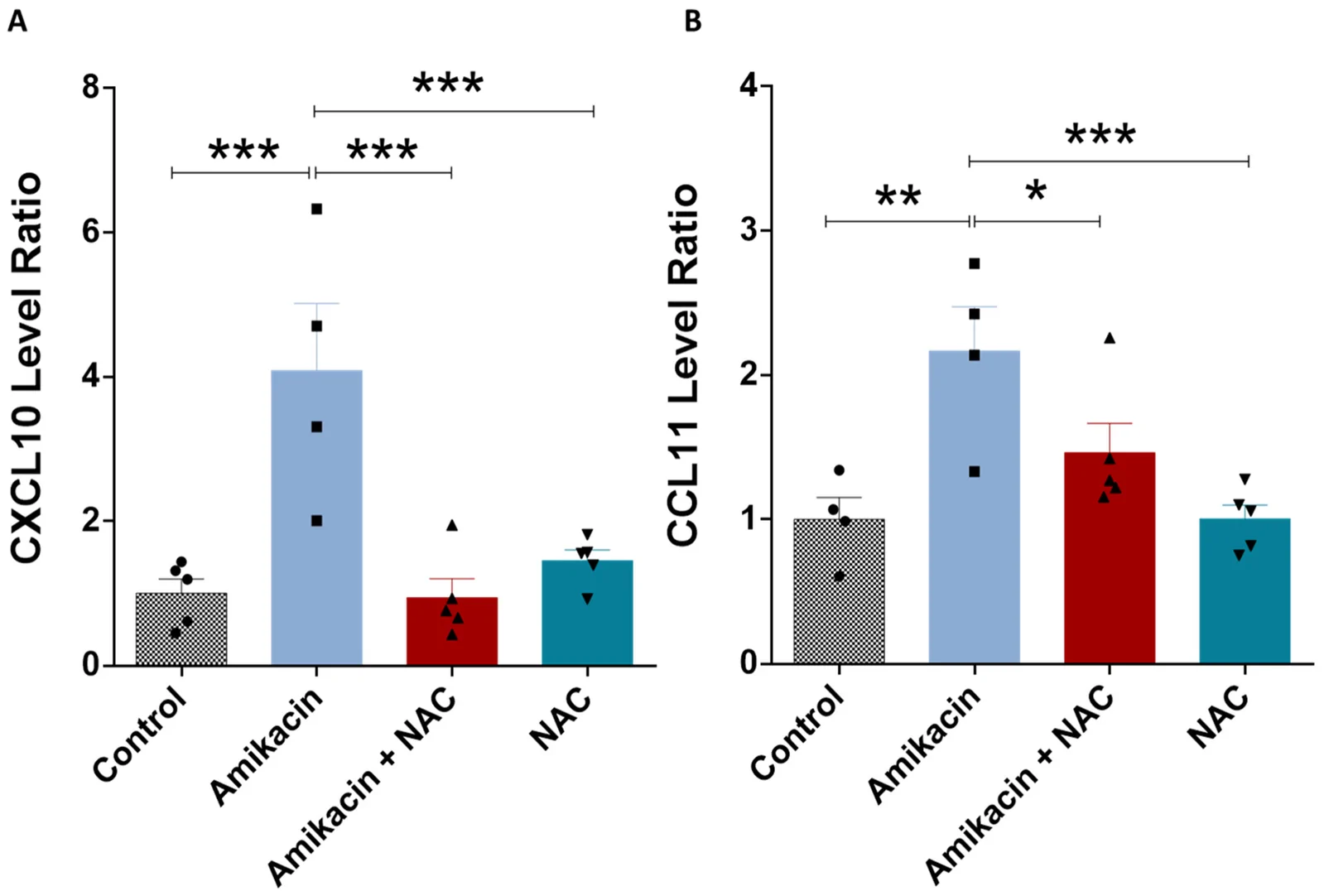
Assessment of pro-inflammatory chemokines. (**A**) The CXCL10 level was significantly higher in the amikacin group as compared to the control, amikacin-NAC, or NAC (*p* < 0.001) groups. (**B**) The CCL11 level was significantly increased in the amikacin group as compared to the control (*p* < 0.01), amikacin-NAC group (*p* < 0.05), and NAC (*p* < 0.001) groups. Data are presented for each treatment group. Data are presented as mean ± SEM (*n* = 4–5/group). * *p* < 0.05; ** *p* < 0.01; *** *p* < 0.001.

One-way ANOVA revealed significant alterations in the serum CXCL10 (F (3, 15) = 10.96, *p* = 0.0005) and CCL11 (F (3, 14) = 7.314, *p* = 0.0035) among the four studied groups. The inflammatory response was characterized by a robust upregulation of two chemokines, indicating a strong signal for leukocyte recruitment. As shown in Figure 12, the chemokines CXCL10 and CCL11 were elevated in the amikacin group. NAC co-administration attenuated the elevation in these two altered chemokines in amikacin-treated mice.

### 3.3. Histopathological and Creatinine Assessment

#### 3.3.1. Renal Cortex and Creatinine

**Figure 13:**
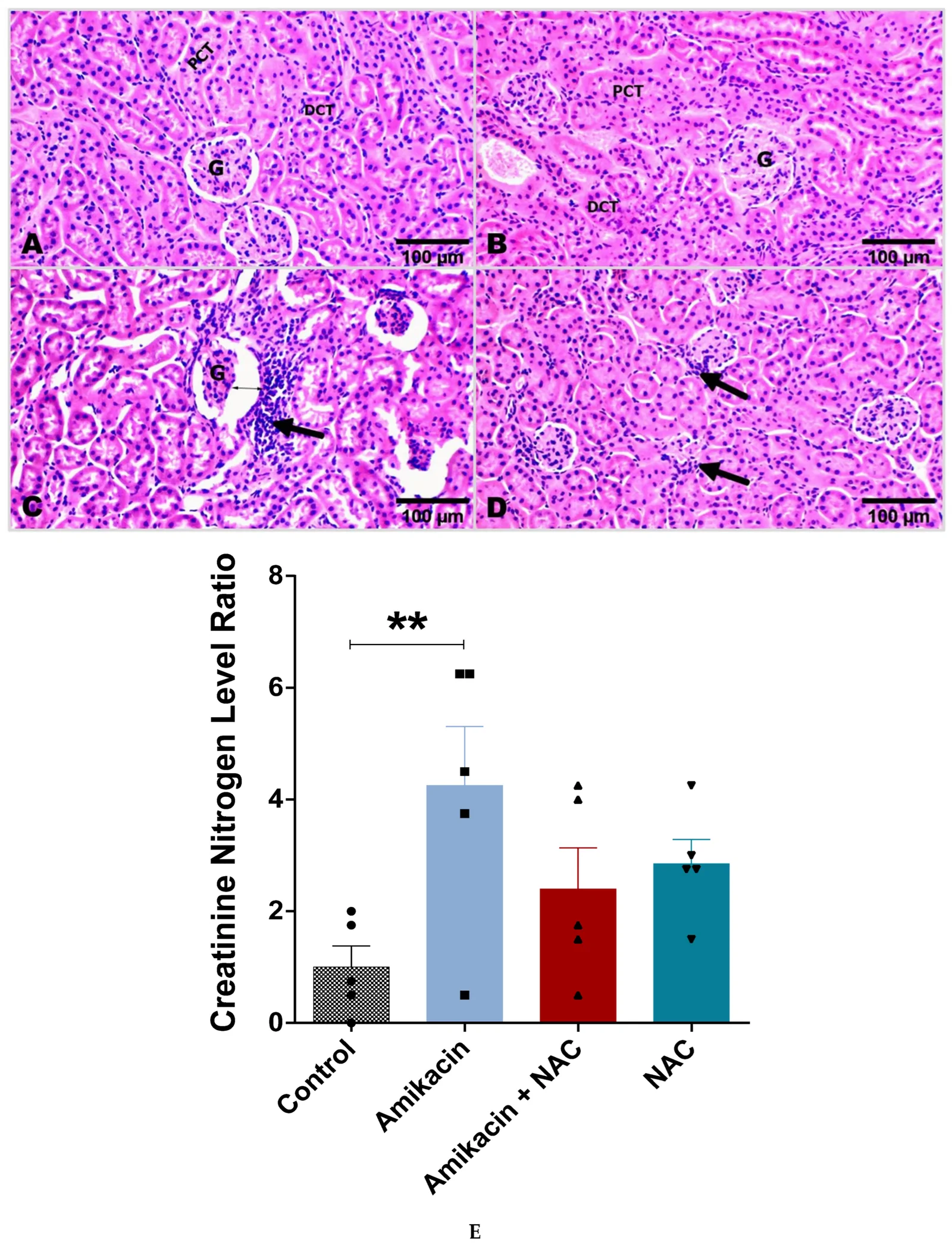
Photomicrographs of animal renal tissue. (**A**) Control renal tissue revealing normal abundant glomerulus (G), proximal convoluted tubules (PCT), and distal convoluted tubule (DCT). (**B**) The NAC-treated group displaying no pathological changes. (**C**) The amikacin-treated group exhibited large aggregation of dark inflammatory cells (arrow), atrophied glomerulus (G), and increases in Bowman’s space (double head arrow). (**D**) Pre-treated group with NAC to amikacin representing marked improvement, limited inflammation (arrows), and improved glomeruli. (H&E-10X- 100 µM). (**E**) The creatinine level was significantly increased in the amikacin group as compared to the control group (*p* < 0.01). Data are presented as mean ± SEM (*n* = 5/group). ** *p* < 0.01.

The control group showed a normal renal cortex structure with abundant glomeruli and convoluted tubules (Figure 13A). In addition, the renal cortex of the NAC-treated group revealed no pathological changes (Figure 13B). Strikingly, administration of amikacin induced renal histopathological alterations, which was evidenced by a large aggregation of dark inflammatory cells, atrophy of the glomerulus, and an expansion of Bowman’s space, suggesting glomerular and interstitial injury (Figure 13C). Furthermore, the renal tissue of the pre-treated group with NAC prior to amikacin displayed a decline in inflammation and preservation of glomerular architecture compared to amikacin-treated group only (Figure 13D). The statistical analysis showed that the creatinine level in the serum was increased with amikacin exposure as compared to the control group, and amikacin-NAC co-exposure or NAC alone did not affect the serum creatinine level as compared to the control or amikacin groups (F (3, 16) = 3.597, *p* = 0.0369) (Figure 13E).

One-way ANOVA showed significant differences in glomerular atrophy (F (3, 16) = 139.4, *p* < 0.0001), interstitial inflammation (F (3, 16) = 80.00, *p* < 0.0001), tubular degeneration (F (3, 16) = 65.27, *p* < 0.0001), tubular dilatation (F (3, 16) = 134.7, *p* < 0.0001), tubular cast formation (F (3, 16) = 121.6, *p* < 0.0001), and the total (F (3, 16) = 643.9, *p* < 0.0001) among the groups. The renal histopathological evaluation revealed that the control and NAC groups maintained a normal architecture without detectable injury. Meanwhile, amikacin administration induced marked pathological alterations, including glomerular atrophy, interstitial inflammation, tubular degeneration, dilatation, and cast formation, reflecting severe renal damage. Pre-treatment with NAC substantially mitigated these alterations except for tubular degeneration, as evidenced by the reduction in glomerular atrophy, tubular cast, and inflammatory changes, suggesting an attenuation of the renal histopathological alterations induced by high-dose amikacin (Table 1).

#### 3.3.2. Cerebral Cortex

**Figure 14:**
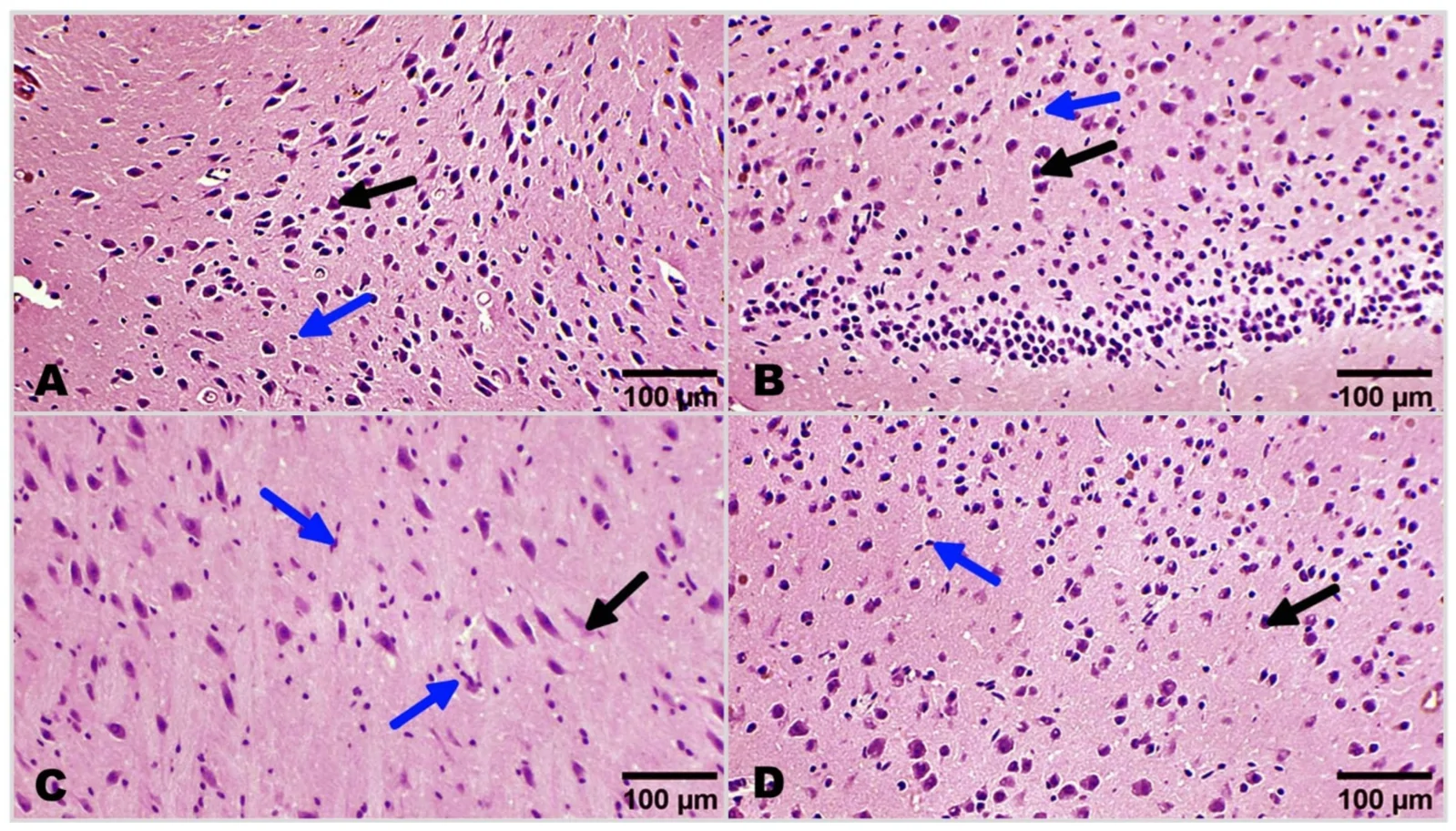
Photomicrographs of cerebral cortex. (**A**) Control cerebral cortex showing a normal structure, with abundant pyramidal neurons (black arrow) and glial cells (blue arrow). (**B**) NAC-treated group revealing no pathological alterations. (**C**) Amikacin-treated group displaying faded and distorted neurons, and gliosis-like changes. (**D**) Pre-treated group with NAC prior to amikacin represented improvements, well-shaped neurons, and lowered gliosis-like changes. (H&E-100X; Scale bar: 100 µm.)

The control cortex maintained its normal laminar organization with abundant, well-defined pyramidal neurons and healthy glial populations (Figure 14A). The NAC-treated group showed a similarly intact architecture (Figure 14B). However, the amikacin-treated cortex exhibited faded, distorted neuronal profiles, reduced cellular clarity, and gliosis-like changes, suggesting drug-induced neuronal stress and degeneration (Figure 14C). Importantly, the group pre-treated with NAC before amikacin exposure demonstrated attenuating effects, with well-shaped neurons, improved cortical morphology, and noticeably gliosis-like changes decline (Figure 14D), suggesting the promising effects of NAC against amikacin-related cortical damage.

One-way ANOVA showed significant differences in neuronal necrosis (F (3, 16) = 54.56, *p* < 0.0001), cortical edema (F (3, 16) = 6.000, *p* = 0.0061), hemorrhagic foci (F (3, 16) = 5.867, *p* = 0.0067), gliosis-like changes (F (3, 16) = 48.00, *p* < 0.0001), and the total (F (3, 16) = 45.76, *p* < 0.0001) among the groups. The histopathological assessment of the cerebral cortex demonstrated that both the control and NAC groups retained a normal cortical architecture without evidence of neuronal damage. Amikacin exposure, however, produced clear neurotoxic alterations characterized by widespread neuronal necrosis, cortical edema, hemorrhagic foci, and gliosis-like changes. Pre-administration of NAC to amikacin markedly attenuated certain pathological changes, with reduced neuronal loss, and less prominent gliosis-like changes, suggesting an attenuation in amikacin-associated cerebral cortical histopathological alterations (Table 2).

## 4. Discussion

Our work highlighted the effects of high-dose amikacin on anxiety-like behavior in the light-dark box and molecular biomarkers and further explored the effects of NAC on selected biochemical, inflammatory, behavioral, and histopathological alterations induced by high-dose amikacin in a preclinical model. The relatively high doses used in this study were intended to establish an experimental toxicity model and do not directly correspond to clinically equivalent human doses. Therefore, caution should be exercised when extrapolating these findings to clinical settings. The results suggested that amikacin might altered behavioral performance in the light-dark box, caused changes in the AST, albumin, and inflammatory profiles, and induced pathological manifestations on renal and cerebral cortex tissues, and several of these alterations were attenuated following NAC pre-treatments. Specifically, co-treatment normalized liver enzymes, albumin, and certain pro-inflammatory cytokines and chemokines. These findings were associated with improved behavioral performance in the light-dark box.

The pharmacological properties of NAC indicate positive therapeutic activities against psychiatric and neurological disorders, with these activities being related to key pathways implicated in anxiety cognition, such as neuroinflammation and glutamate homeostasis [20,29]. In the present work, exposure to a high dose of amikacin was associated with observed anxiety behavior in the light-dark box suggestive of anxiety-like behavior, which were ameliorated by NAC co-administration. It has previously been shown that acute hepatic encephalopathy likewise induces anxiety behaviors [30] and that in adult male rats, anxiety caused by interleukin-1β can be reduced by treatment with the peripheral anti-inflammatory cytokine interleukin-10 [31]. A limitation of the present study is that locomotor activity was not independently assessed. Therefore, although the light-dark box findings are suggestive of anxiety-like behavior, reduced exploration may also reflect general sickness behavior, malaise, sedation, auditory alterations, or decreased locomotor activity induced by amikacin treatment. Our findings suggest that NAC attenuated anxiety-like behavior observed in the light-dark box. Whether these changes are mechanistically related to alterations in systemic cytokines and chemokines remains to be determined. While no formal randomized controlled trials investigating primary anxiety conditions are available at present, the combination of mechanistic, translational, and clinical data obtained from psychiatric populations indicates a physiologically plausible anxiolytic and neuroprotective activity of NAC [32,33,34]. In particular, as an important cysteine donor for glutathione synthesis, NAC can help restore redox balance and possibly prevent synaptic damage [35,36].

With regard to the liver, therapeutic and translational studies have demonstrated that NAC can restore hepatic glutathione, free radical scavenging, and lipid peroxidation; it can also attenuate hepatocyte necrosis and apoptosis, and stabilize both transaminases (AST, ALT) and cholestatic markers (ALP, bilirubin) [37,38,39,40]. The ability of NAC to preserve mitochondrial membrane potential, hinder the mitochondrial permeability transition and increase NRF2-dependent antioxidant enzymes further bolsters hepatocyte resilience to oxidative loading [38]. In a prospective study on patients with liver cirrhosis, treatment with NAC produced decreases in ALT, AST, and ALP compared to baseline, indicating less hepatocellular and cholestatic injury [41]. Along with our findings, these data are consistent with the observed attenuation of AST and ALT elevations in animals exposed to high-dose amikacin. Although it is stated in the literature that NAC reduces amikacin-induced nephrotoxicity [23], we did not observe significant between-group differences in blood urea nitrogen or uric acid. Importantly, we found that creatinine level was increased in the amikacin group as compared to the control group, which was not significantly improved by NAC pre-treatment. However, there are not significant changes in creatinine levels between controls and amikacin-NAC groups. Although creatinine was not significantly improved by NAC, histopathological evaluation suggested an attenuation of renal cortical structural alterations following NAC and amikacin co-administration. Therefore, NAC attenuated selected renal histopathological alterations but did not demonstrate complete recovery of renal function. Similarly, reductions in ALT and AST reflect changes in selected hepatic biomarkers rather than definitive hepatoprotection. Future studies should include sensitive renal markers such as KIM-1, NGAL, and cystatin C.

NAC may also reduce levels of lactate dehydrogenase (LDH), a marker for cellular impairment and membrane integrity [42]. In preclinical liver models, NAC has been shown to attenuate LDH release following ischemia-reperfusion [42]. In acute pancreatitis, NAC attenuates the elevation in liver enzymes and LDH amylase, suggesting less hepatocellular necrosis and release of LDH [43]. In NAFLD models, NAC suppresses lipotoxic and oxidative stress, which is associated with improved levels of pro-inflammatory cytokines [44]. NAC also stabilizes mitochondrial membranes and prevents the mitochondrial permeability transition, thereby maintaining cellular integrity and decreasing the apoptotic and necrotic cell death associated with LDH release [45]. In clinical settings including obstructive jaundice surgery, NAC administration is associated with improvements in organ function, particularly improved liver function [39]. In our present study, although the statistical analysis did not show differences among the groups, the amikacin group exhibited higher LDH compared to controls, whereas the co-treated NAC-amikacin group had a profile, which was not significantly different from the control group. Future studies are warranted to explore the role of NAC in modulating serum LDH in diseases involving impaired inflammatory signaling.

A previous study reported that amikacin, especially at high dose, might cause cytotoxic effects through influencing pro-inflammatory cytokines [46]. NAC can decrease NF-κB activation, decrease pro-inflammatory cytokines and facilitate mitochondrial efficiency [47,48,49]. In our study, several major pro-inflammatory cytokines were elevated during amikacin administration, including IL-6, IL-18, IL-22, and IL-12p70. Co-treatment with NAC consistently attenuated these elevated biomarkers, maintaining systemic cytokine levels near those observed in controls. In addition to cytokines, NAC has previously been demonstrated to decrease inflammatory chemokines in tissues such as smooth muscle and the human kidney [50,51]. Here, we observed NAC to exert inhibitory effects against chemokines such as CXCL10 and CCL11, indicating concurrent alterations in peripheral inflammatory mediators together with biochemical changes. The antioxidant and anti-inflammatory mechanisms of NAC remain hypothetical, as oxidative stress and NF-κB/NRF2 pathways were not directly assessed. Circulating cytokines also cannot establish tissue-specific inflammation. Future studies should directly investigate these mechanisms and tissue-specific inflammatory markers.

## 5. Conclusions

**Figure 15:**
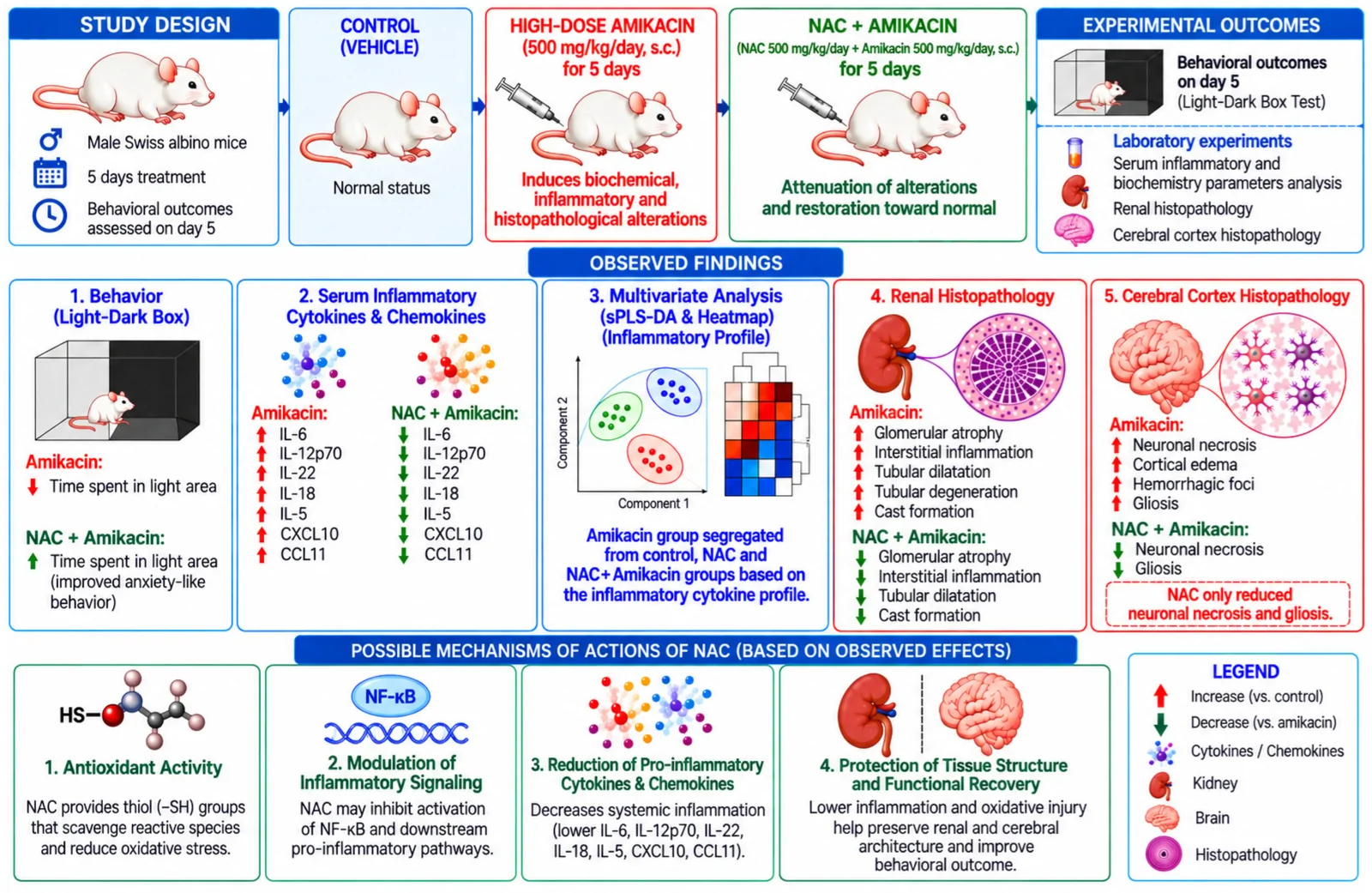
Graphical summary of the observed effects of *N*-acetylcysteine (NAC) in mice exposed to high-dose amikacin. Mice received amikacin (500 mg/kg/day) with or without NAC (500 mg/kg/day) for 5 days, and behavioral outcomes were assessed on day 5. High-dose amikacin reduced time spent in the light compartment, increased serum ALT and AST, elevated inflammatory cytokines and chemokines (IL-5, IL-6, IL-12p70, IL-22, IL-18, CXCL10, and CCL11), and induced renal and cerebral cortical histopathological alterations. NAC co-treatment attenuated the elevations in liver enzymes and inflammatory mediators, improved light-dark box performance, reduced renal glomerular atrophy, interstitial inflammation, tubular dilatation, and cast formation, and reduced neuronal necrosis and gliosis-like changes in the cerebral cortex. The lower panel illustrates possible mechanisms of NAC action, including antioxidant activity and modulation of inflammatory signaling, which may contribute to the observed reductions in inflammatory mediators and tissue injury.

High-dose amikacin exposure was associated with certain biochemical, inflammatory, and histopathological alterations in the experimental model as reported in our study. These behavioral impairments observed in the light-dark assay were accompanied by alteration of AST, albumin, elevation in serum inflammatory cytokines and chemokines, gliosis-like changes, and glomerular and interstitial injury. These findings suggest that high-dose amikacin exposure is associated with alterations in certain inflammatory mediators and biochemical markers, as well as renal and cerebral cortex morphology. However, future follow-up studies should incorporate specific neuronal markers, particularly NeuN, to confirm and further characterize the neuronal alterations identified by H&E staining and to provide more specific evidence regarding the effects of high-dose amikacin on the cerebral cortex and the potential specific effects of NAC. In the present study, high-dose amikacin exposure was associated with increased levels of certain inflammatory markers. Our findings suggested that amikacin-induced toxicity may involve alterations in immune mediators, chemokine expression, and inflammatory signaling pathways. Although the present data demonstrate concurrent changes in inflammatory, biochemical, and behavioral parameters, they do not establish causal relationships among these outcomes. Therefore, it is plausible that inflammatory processes contribute to the observed alterations; however, the underlying mechanisms require further investigation. Several of the measured biochemical, inflammatory, behavioral, and histopathological alterations were attenuated following NAC treatment (Figure 15).

Future studies are warranted to explore the degree to which amikacin toxicity involves mitochondrial dysfunction, transporter-mediated drug accumulation, and inflammation and oxidative stress. In addition, the future studies should investigate the effects of amikacin toxicities on oxidative stress-related parameters, including glutathione, reactive oxygen species, lipid peroxidation, and antioxidant enzymes. In line with our findings, it also remains to be determined whether NAC treatment may contribute to protecting the immune system, preventing organ damage, and restoring physiologic homeostasis. Therefore, the present findings should be considered preliminary and require confirmation in future studies with larger sample sizes. Moreover, further studies using clinically relevant exposure levels, pharmacokinetic assessments, and both prophylactic and therapeutic NAC regimens are warranted before clinical applicability can be determined.

## Figures and Tables

**Table 1:** Histopathological scoring of the renal cortex in the control, NAC, amikacin, and amikacin + NAC groups of mice. One-way ANOVA showed significant differences in glomerular atrophy, interstitial inflammation, tubular degeneration, tubular dilatation, and cast formation among the groups. Post-hoc analysis revealed an increase in glomerular atrophy, interstitial inflammation, tubular degeneration, dilatation, and cast formation as compared to in the control or NAC groups, effects reversed by NAC pre-treatments except for tubular degeneration.

Groups	Glomerular Atrophy	Interstitial Inflammation	Tubular Cast	Tubular Degeneration	Tubular Dilatation	Total
**Control**	0	0	0	0	0	0
**NAC**	0	0	0	0	0	0
**Amikacin**	2.7 ± 0.1 ^a #, b #^	2.5 ± 0.2 ^a #, b #^	1.3 ± 0.1 ^a #, b #^	1.7 ± 0.2 ^a #, b #^	2.6 ± 0.2 ^a #, b #^	10.8 ± 0.4 ^a #, b #^
**Amikacin + NAC**	1.6 ± 0.2 ^a #, b #, c #^	1.5 ± 0.2 ^a #, b #, c #^	1 ± 0 ^a #, b #, c **^	1.4 ± 0.1 ^a #, b #^	1 ± 0 ^a #, b #, c #^	6.6 ± 0.2 ^a #, b #, c #^

^a^, compared to the control group; ^b^, compared to the NAC group; ^c^, compared to the amikacin group. Data are presented as mean ± SEM. ** *p* < 0.01; # *p* < 0.0001.

**Table 2:** Histopathological scoring of the cerebral cortex in the control, NAC, amikacin, and amikacin + NAC groups of mice. One-way ANOVA showed significant differences in neuronal necrosis, cortical edema, hemorrhagic foci, and gliosis-like changes among the groups. Post-hoc analysis revealed an increase in neuronal necrosis, cortical edema, hemorrhagic foci, and gliosis-like changes as compared to the control and NAC groups. NAC pre-treatments reversed amikacin-induced neuronal necrosis and gliosis-like changes.

Groups	Neuronal Necrosis	Cortical Edema	Hemorrhagic Foci	Gliosis-like Changes	Total
**Control**	0	0	0	0	0
**NAC**	0	0	0	0	0
**Amikacin**	2.6 ± 0.2 ^a #, b #^	1 ± 0.3 ^a **, b **^	0.8 ± 0.2 ^a **, b **^	2.4 ± 0.2 ^a #, b #^	6.8 ± 0.7 ^a #, b #^
**Amikacin + NAC**	1.6 ± 0.2 ^a #, b #, c ***^	0.6 ± 0.2 ^a *, b *^	0.4 ± 0.2	1.6 ± 0.2 ^a #, b #, c **^	4.2 ± 0.6 ^a #, b #, c **^

^a^, compared to the control group; ^b^, compared to the NAC group; ^c^, compared to the amikacin group. Data are presented as mean ± SEM. * *p* < 0.05, ** *p* < 0.01; *** *p* < 0.001; # *p* < 0.0001.

## Data Availability

The original contributions presented in this study are included in the article/Supplementary Materials. Further inquiries can be directed to the corresponding author.

## Supplementary Materials

The following supporting information can be downloaded at: https://www.mdpi.com/article/10.3390/biology15181618/s1, Table S1: Raw *p*-values and Benjamini–Hochberg false discovery rate (FDR)-adjusted p-values for the 20- plex cytokine/chemokine panel.
