# Supplementary material for: N-Acetylcysteine Attenuates Amikacin-Induced Biochemical, Inflammatory, and Histopathological Alterations in Mice
Source: Biology (Basel). 2026 Sep 14;15(18):1618. doi: 10.3390/biology15181618 (PMC13604663; doi:10.3390/biology15181618)
Supplement: Supplementary file 1 [file biology-15-01618-s001.zip › biology-4530924-supplementary.pdf]

Supplementary Table S1. Raw *p*-values and Benjamini–Hochberg false discovery rate (FDR)-adjusted *p*-values for the 20- plex cytokine/chemokine panel.

**Rank**	**ID**	**Original *p*-values**	**Benjamini-Hochberg Adjusted *p*-values**	**Significant using an FDR of 0.05**
1	CXCL10	0.0005	0.005	Yes
2	IL-12P70	0.0005	0.005	Yes
3	IL-5	0.0009	0.006	Yes
4	IL-22	0.0024	0.01	Yes
5	IL-18	0.0025	0.01	Yes
6	CCL11	0.0035	0.011666666666666667	Yes
7	IL-6	0.0098	0.028	Yes
8	TNF-ALPHA	0.0215	0.053749999999999999	No
9	CCL7	0.0353	0.078444444444444444	No
10	IL-17A	0.1578	0.3156	No
11	CXCL1	0.2172	0.3949090909090909	No
12	IL-27	0.3281	0.5141538461538462	No
13	CCL5	0.3342	0.5141538461538462	No
14	CCL2	0.3781	0.517375	No
15	CXCL2	0.4075	0.517375	No
16	CCL3	0.4139	0.517375	No
17	IL-23	0.4844	0.5413333333333333	No
18	IL-13	0.4872	0.5413333333333333	No
19	IL-2	0.712	0.7494736842105263	No
20	GM-CSF	0.7643	0.7643	No
